# Pharmacogenetics Role of Genetic Variants in Immune-Related Factors: A Systematic Review Focusing on mCRC

**DOI:** 10.3390/pharmaceutics14112468

**Published:** 2022-11-15

**Authors:** Lucia Scarabel, Alessia Bignucolo, Giuseppe Toffoli, Erika Cecchin, Elena De Mattia

**Affiliations:** Experimental and Clinical Pharmacology, Centro di Riferimento Oncologico di Aviano (CRO) IRCCS, via Franco Gallini n. 2, 33081 Aviano, Italy

**Keywords:** colorectal cancer, mCRC, biomarkers, genetic susceptibility factors, immunotherapy, pharmacogenetics, precision medicine, personalized medicine, immune system, clinical implementation

## Abstract

Pharmacogenetics plays a key role in personalized cancer treatment. Currently, the clinically available pharmacogenetic markers for metastatic colorectal cancer (mCRC) are in genes related to drug metabolism, such as DPYD for fluoropyrimidines and UGT1A1 for irinotecan. Recently, the impact of host variability in inflammatory and immune-response genes on treatment response has gained considerable attention, opening innovative perspectives for optimizing tailored mCRC therapy. A literature review was performed on the predictive role of immune-related germline genetic biomarkers on pharmacological outcomes in patients with mCRC. Particularly, that for efficacy and toxicity was reported and the potential role for clinical management of patients was discussed. Most of the available data regard therapy effectiveness, while the impact on toxicity remains limited. Several studies focused on the effects of polymorphisms in genes related to antibody-dependent cellular cytotoxicity (FCGR2A, FCGR3A) and yielded promising but inconclusive results on cetuximab efficacy. The remaining published data are sparse and mainly hypothesis-generating but suggest potentially interesting topics for future pharmacogenetic studies, including innovative gene–drug interactions in a clinical context. Besides the tumor immune escape pathway, genetic markers belonging to cytokines/interleukins (IL-8 and its receptors) and angiogenic mediators (IGF1) seem to be the best investigated and hopefully most promising to be translated into clinical practice after validation.

## 1. Introduction

Colorectal cancer (CRC) is the third most frequent cancer and the second leading cause of death in the world [1]. Further, 20% of the cases have a metastatic CRC (mCRC) at diagnosis and, among the remaining non-metastatic patients, it was observed a 30% probability to develop metastasis during the treatment [2]. 5-fluorouracil (5-FU) in combination with leucovorin (LV) remains the cornerstone of most chemotherapeutic schedules used to treat advanced CRC. The combination of 5-FU/LV with either oxaliplatin (e.g., FOLFOX) or irinotecan (e.g., FOLFIRI) represents a well-established standard first-line treatment [3,4]. Capecitabine could be efficaciously used in place of 5-FU/LV association in several regimens, as XELOX (capecitabine and oxaliplatin) and XELIRI (capecitabine and irinotecan) [4]. A quartet combination FOLFOXIRI (5-FU, LV, oxaliplatin, irinotecan) was also positively tested. In the last decade, the treatment of mCRC has further achieved great advances with the development of biological agents targeting the vascular endothelial growth factor (VEGF, i.e., bevacizumab, aflibercept, ramucirumab) and epidermal growth factor receptor (EGFR, i.e., cetuximab, panitumumab) cascade or leading to a multiple-kinase inhibition (regorafenib) [4]. In the recent period, immunotherapies have also been considered in specific mCRC molecular subtypes [5]. Particularly, regulatory agencies (the Food and Drug Administration (FDA) and the European Medicines Agency (EMA)) approved pembrolizumab for the treatment of metastatic and unresectable microsatellite instability-high (MSI-H)/mismatch repair deficiency (dMMR) CRC, as well as nivolumab plus ipilimumab for the treatment of adult and pediatric patients aged 12 years and older with MSI-H/dMMR mCRC after progression following the use of fluoropyrimidine, oxaliplatin, and irinotecan. The introduction of the immunocheckpoint inhibitors (ICI) has revolutionized the management and survival of many mCRC patients with highly immunogenic characteristics such as MSI-H features. Immunotherapy could be adopted to boost the immune response in the body and/or suggest to immune cells how to identify and destroy cancer cells in the tumor microenvironment (TME), ameliorating the clinical outcome of these patients.

**Figure 1:**
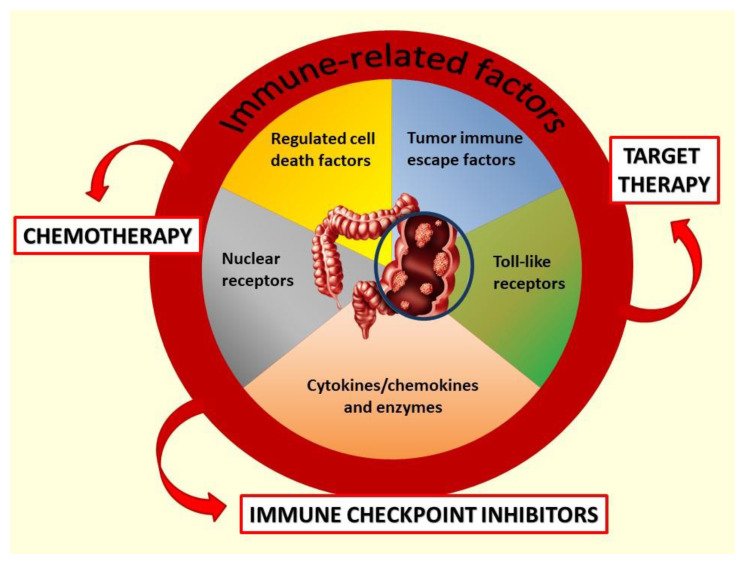
The interplay between immune-related factors and pharmacological strategies to fight mCRC.

Chronic inflammation and host immune system dysfunction are well-recognized important factors that contribute to CRC development, progression, and prognosis [6,7,8,9]. Based on molecular features including immune-related aspects, four different consensus subtypes (CMSs) with different clinical implications have been determined for CRC: the highly immunogenic known as “immune” (CSM1), the inflamed immune-suppressive known as “mesenchymal” (CSM4), and other two poorly immunogenic subtypes called “canonical” (CSM2) and “metabolic” (CSM3) [10]. The cancer-related inflammatory and immune response has been also reported to play a crucial role in the modulation of the efficacy of mCRC treatment, not only concerning ICI but also to standard chemotherapy and targeted agents (Figure 1).

Inflammation and the immune system were indicated to impact chemotherapy effectiveness by multiple different mechanisms, including the modulation of chemotherapy-mediated tumor cell death and regulation of inflammatory-related transcriptional factors that in turn affect the expression of absorption, distribution, metabolism, and excretion (ADME) genes [11,12,13]. Moreover, the same chemotherapeutics, including irinotecan, 5-FU, and oxaliplatin, were indicated to display an immune-modulator effect, influencing the overall antitumor response and disease outcome [14,15,16,17,18]. The immune response has been reported to be important also in determining the cytotoxicity of some targeted agents such as cetuximab. Another mechanism of action of the anti-EGFR drug, beyond the EGFR blockade, is antibody-dependent cellular cytotoxicity (ADCC), also referred to as antibody-dependent cell-mediated cytotoxicity [18,19,20]. The cetuximab structure exhibits not only the antigen-binding but also a crystalline fragment (Fc fragment), enabling it to bind to the fragment Cγ receptor (FcγR) of immune cells, including the natural killer (NK). The binding of cetuximab to Fc fragment triggers the ADCC pathway.

Despite favorable therapeutic results in recent years, a significant inter-individual heterogeneity in therapy outcome still constitutes a critical problem in mCRC management. Moreover, with the increasing number of effective drugs for mCRC patients often used in combination, the selection of the more appropriate first-line therapeutic options becomes a complex issue influencing the course of therapy [21]. Therefore, the definition of molecular markers that predict which patients will benefit from a specific treatment option could significantly impact the clinical decision-making and therapeutic planning. Some molecular characteristics of tumors (i.e., KRAS, NRAS, and BRAF mutations; MSI-H/dMMR status) [21,22,23,24], as well as clinical features (i.e., right- and left-sided mCRC) [21,25], have been identified as predictors of therapy outcome and patient prognosis. However, a significant variability in the response to treatment is still present and additional markers should be defined. On this ground, the evaluation of the host genetic profile could contribute to predict the chemo-responsiveness and to better stratify patients who undergo therapy for mCRC based on the treatment outcome [26]. The emerging role of inflammation and the immune system in mCRC treatment opens a novel field for pharmacogenetic studies aimed at defining the role of host variability in inflammation and immune-related genes in predicting treatment response and patient outcome. In particular, the study of genetic variants in biomarkers, involved in the acute phase, innate, and adaptive immune responses, provides a better understanding of the role of immune regulation in cancer and thus its influence not only on the efficacy and toxicity of chemotherapy, but also on the mechanisms of resistance to therapy. The aim of this review is to critically report and discuss the current literature on the effect of inflammation and immune-related germline variants as predictive markers of mCRC systemic therapy outcome and how they can help stratify patients according to the toxicity risk as well as the likelihood to benefit from the administration of specific anti-tumor agents.

## 2. Methods

**Figure 2:**
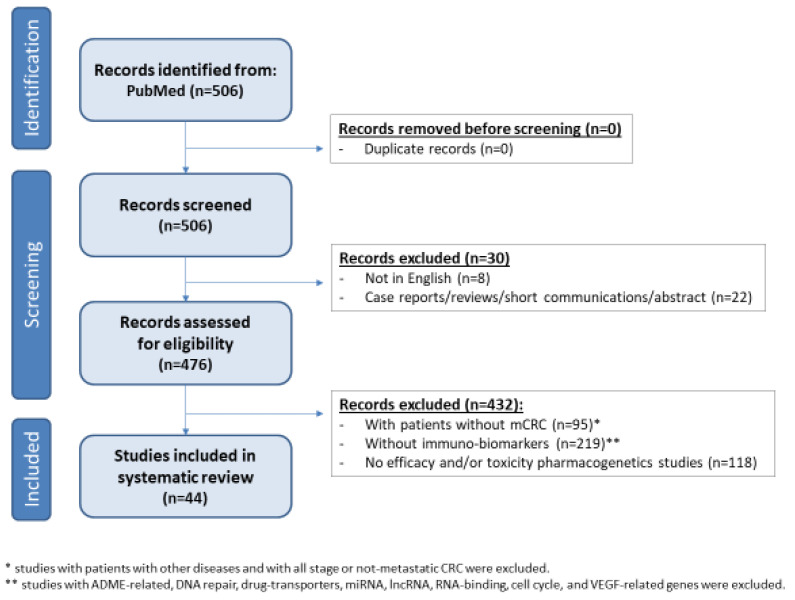
Studies selection flow diagram.

This work used a systematic review methodology and adheres to the Preferred Reporting Items for Systematic Reviews and Meta-Analysis (PRISMA) guidelines. The selected papers regard the predictive role in terms of efficacy and/or toxicity of genetic variants in immune-related biomarkers in patients with mCRC. The systematic literature search was conducted on 21 October 2021 using PubMed database. Boolean operators AND/OR were used to combine search terms. The search strategy included key terms as follows: (((“colorectal cancer” OR CRC) AND (patient OR patients) AND (immun*) AND (therapy OR chemotherapy OR treatment* OR leucovorin OR oxaliplatin OR irinotecan OR fluoropyrimidine OR 5-FU OR *fluorouracil* OR 5FU OR capecitabine OR cetuximab OR bevacizumab OR aflibercept OR ramucirumab OR regorafenib OR panitumumab OR ipilimumab OR nivolumab OR pembrolizumab OR trifluridine OR tipiracil OR encorafenib OR TAS-102 OR vemurafenib OR pertuzumab OR trastuzumab OR lapatinib OR deruxtecan OR sotorasib OR adagrasib) AND (polymorphism* OR pharmacogenetic* OR pharmacogenomic*)) NOT ((review[Publication Type]) OR (“systematic review”[Publication Type]))). Only manuscripts published in English were considered, and systematic reviews, reviews, short communications, abstracts and case reports were excluded after a systematic screening. Only studies with metastatic patients and immune-related biomarkers, defined as acute-phase cytokines and enzymes, angiogenic mediators, nuclear receptors, toll-like receptors, cytokines and chemokines activators in the early phase of immune response, tumor immune escape factors, cytokines and chemokines activators in the late phase of immune response, cell death regulation factors were considered eligible. Studies with ADME-related, DNA repair, drug-transporters, miRNA, long non-coding RNA (lncRNA), RNA-binding, cell cycle, VEGF-related genes were excluded. In particular, all the inherited genetic variants with a predictive role in terms of efficacy measured as overall survival (OS), progression-free survival (PFS), overall response rate (ORR), response rate (RR), disease control rate (DCR) and/or toxicity measured as any toxicities, hematotoxicity, diarrhea, gastrointestinal, skin rash toxicity were included in the systematic review based on immune system factors acting in the acute-phase/innate, and adaptive immune response (Figure 2), and their possible role in clinical management was discussed. This systematic research was performed independently by two authors (A.B. and L.S.).

## 3. Results

### 3.1. Studies Selection

A total of 506 records were found running the search strategy on 21 October 2022. No duplicate was found, After the first screening evaluating the language and the type of records, 30 records were excluded. All the remaining 476 papers were screened by title, abstract, and full text to identify the eligible studies. 432 records were excluded because not fit with the eligibility criteria. A total of 44 manuscripts were considered and discussed in the systematic review (Figure 2).

### 3.2. Summary of Evidences

All the genes and their inherited genetic variants with a predictive role in terms of efficacy and/or toxicity retrieved from the 44 studies highlighted by the systematic review are summarized in Table 1. These results are subdivided in eight groups: (1) acute-phase cytokines and enzymes, (2) angiogenic mediators, (3) nuclear receptors, (4) toll-like receptors, (5) cytokines and chemokines activators in the early phase of immune response, (6) tumor immune escape factors, (7) cytokines and chemokines activators in the late phase of immune response, (8) cell death regulation factors. Moreover, to highlight the risk of bias of the results, four levels of evidences (high, moderate, low, very low) were adopted. First of all, the study design was evaluated, attributing “high” level of evidence to the meta-analysis and randomized controlled studies, a “moderate” level to training/replication/control studies, a “low” level to observational studies. Then, other aspects including low sample size, heterogenicity in patients’ ethnicity, in treatment used, and in results of different studies were considered to downgrade the level of evidence.

## 4. Discussion

### 4.1. Acute-phase Cytokines and Enzymes

Interleukin-1β (IL-1β), interleukin-6 (IL-6), and tumor necrosis factor-α (TNF-α) are pyrogenic cytokines with a role in the acute-phase response. Under hypoxic states, IL-6 promotes hypoxia-inducible factor 1α (HIF-1α, *HIF1A*) and signal transducer and activator of transcription-3 (STAT-3) transcription, which stimulates VEGF expression, blood vessel formation, and tumor growth through defective angiogenesis. In a total of 775 mCRC patients, 511 patients were treated with first-line FOLFIRI plus bevacizumab (223 in training and 288 in validation cohorts) and 264 with FOLFIRI plus cetuximab (control cohort), polymorphisms in the IL-6/STAT-3 pathway were investigated in the development, invasion, and spreading of mCRC due to their role in facilitating immune tolerance within the TME [27]. Patients carrying the *IL6*-rs2069837-G allele had shorter median progression-free survival (PFS) compared with the AA genotype both in the training and validation cohorts (Hazard Ratio (HR):1.50; *p* = 0.033; HR:1.34, *p* = 0.047, respectively) [27]. Moreover, patients with the *IL6*-rs2069837-AA genotype had higher tumor response (67% AA vs. 52% any G, *p* = 0.026) only in the validation cohort. The *IL6*-rs2069837-G allele leads to increase IL-6 expression and was supposed that it serves as a surrogate for resistance towards anti-VEGF therapy in mCRC patients [27,90].

The inflammatory-related transcription factor, STAT-3, is hyper-activated in several human cancers, leading to proliferation, apoptosis, division, and differentiation of tumor cells. Usually, prolonged activation of the IL-6/STAT-3 signal may result in poor therapy outcomes as well as drug-related side effects via alterations of drug bioavailability [91]. Altered expression of STAT-3 deriving from polymorphic variants may predispose patients administered irinotecan-based chemotherapy to gastrointestinal (GI) epithelium damage and consequent side effects (i.e., mucositis, diarrhea). *STAT3*-rs1053004 polymorphism is located in the 3′UTR region and regulates the protein expression by altering its transcriptional activity [92]. In this regard, a discovery/replication study in 400 mCRC patients treated with first-line FOLFIRI, highlighted the protective role of the *STAT3*-rs1053004-C allele against grade 3-4 GI toxicity (OR:0.51, *p* = 0.045; OR:0.39, *p* = 0.043, respectively, in the discovery and replication groups) [29].

The prolonged inflammatory effects of IL-1β are counterbalanced by the action of the interleukin-1 receptor antagonist (IL-1RA) cytokine that competitively inhibits the binding of circulating IL-1β during an inflammatory event to quench acute inflammation and avoid tumor angiogenesis/metastasis. Two polymorphisms in the *IL-1RA* gene (rs4251961 and rs579543) influencing IL-1RA circulating levels may alter this mechanism. The highest IL-1RA production was reported in carriers of the homozygous *IL-1RA*-rs4251961-TT and *IL-1RA*-rs579543-TT genotypes. In 180 mCRC patients treated with second-line irinotecan or oxaliplatin-based regimens ± cetuximab, carriers of at least one T allele for these two polymorphisms had a favorable survival profile (risk ratio:0.64, *p* = 0.018) [93].

Prostaglandin-endoperoxide synthase 2 (PTGS2, also known as cyclooxygenase 2 [COX-2]) is the key enzyme in inflammatory prostaglandin biosynthesis in many cells, including colonic epithelial cells, and it is induced in response to inflammatory stimuli. Pro-inflammatory prostaglandin E2 (PGE2) is a potent inflammatory lipid mediator that is generated by COX-2 conversion of arachidonic acid, representing the main prostaglandin in CRC [94]. The alterations in multiple pathways, including PTGS2, EGFR, as well as RAS, are significantly involved in CRC development and progression. The cross-talk between pathways of inflammatory response/prostaglandins biosynthesis and EGFR signaling plays a key role in enhancing the growth and spread of tumor cells [94,95]. Particularly, during the inflammatory response, PTGS2 is involved in the processes of vasoconstriction, vasodilatation, platelet aggregation, and immunomodulation. Prostaglandins and other lipid mediators of inflammation are produced quickly by the macrophages and then their actions are followed by those of cytokines such as IL-6 and interleukin-10 (IL-10) with the consequent activation of the EGFR signal, promoting the proliferation of tumor cells [94,96]. Concerning response to anti-EGFR agents, *PTGS2*-rs5275 and *PTGS2*-rs20417 polymorphisms have raised the interest of researchers as potential predictor biomarkers of efficacy [31,33]. The rs20417 polymorphism is located in the 3′UTR region of the *PTGS2* gene, and the minor C allele has been associated with a decrease in promoter activity, whereas the minor C allele of the rs5275 polymorphism in exon 10 decreases mRNA stability [95]. In 76 mCRC patients treated with XELOX chemotherapy, the *PTGS2*-rs5275-TT genotype was correlated with better PFS and OS (HR:0.47, *p* = 0.046, and HR:0.16, *p* = 0.013, respectively) [32]. Contrariwise, in the study by Lurje et al. involving 130 mCRC patients treated with cetuximab, *PTGS2*-rs20417-CC and *PTGS2*-rs5275-CC genotypes were associated with longer PFS in univariate analysis (relative risk (RR):0.31, *p* = 0.032 and RR:0.67, *p* = 0.0003, respectively). *PTGS2*-rs5275 maintained significance also in multivariate analysis (RR:0.53; *p* = 0.013) [31]. Conflicting data also come from a large study of 815 Caucasian mCRC patients treated with cetuximab-based therapy in which the *PTGS2*-rs5275 polymorphism did not emerge as a predictive marker of efficacy for the therapy [33]. Further studies are certainly needed to clarify the actual role of these polymorphisms in the response to anti-EGFR treatment. The impact that such polymorphisms have on both gene expression and COX-2 activity may help to better explain the resistance to targeted therapies that sometimes occur in a TME in which the immune response to cancer cells appears to be impaired.

In addition, genes involved in the cyclic GMP–AMP synthase (cGAS)–stimulator of the interferon genes (STING) pathway have an important role in the early phase of immune response by inducing type-I interferon (IFN) [97]. In a case/control study of 451 Caucasian mCRC patients, 129 treated with FOLFIRI+cetuximab and the others with FOLFIRI+bevacizumab as controls, the genes in the cGAS/STING pathway were evaluated.

In the FOLFIRI+cetuximab group, patients with *STING*-rs7380824-any T allele showed a lower ORR (any T vs. CC: 60.7% vs. 81.5%, *p* = 0.04), while patients with *STING*-rs11311769-any T allele had a shorter OS (HR:2.98; *p* = 0.0085) [34]. These polymorphisms are missense variants (rs7380824: R293Q; rs11311769: R232H) and their variants can determine a reduction in the level of IFN production and then in the innate immune response [98]. In the FOLFIRI-cetuximab group, another variant related to INF emerged as significant: the *IFNB1*-rs1051922 polymorphism that affects the IFN-β production due to a premature stop codon. The presence of *IFNB1*-rs1051922-GA and AA genotypes was correlated with shorter PFS (HR:2; *p* = 0.02; HR:2.19, *p* = 0.02, respectively) [34]. In the FOLFIRI+bevacizumab group, the cGAS-rs6907936 polymorphism emerged as significant; the presence of any G allele showed higher ORR (81.5% vs. 58.4%, *p* = 0.04) [34]. The mechanism of action of the antiangiogenic drug bevacizumab seems less likely to directly impact the immune system by ADCC; however, an indirect effect on tumor vasculatures due to the STING/cGAS pathway could interfere with the bevacizumab antitumor activity. Nevertheless, more studies are needed to confirm this hypothesis.

### 4.2. Angiogenic Mediators

Inflammation can cause endothelium changes, known as endothelial cell activation, which allow it to participate in the inflammatory response [99,100]. The immune-related core changes of endothelial cell activation include cytokine production, expression of leukocyte adhesion molecules, and up-regulation of human leukocyte antigen (HLA) molecules, then determining neutrophil recruitment [99].

The C-X-C motif chemokine ligand 12 (CXCL12)/C-X-C chemokine receptor type 4 (CXCR4) axis, mainly activated by VEGF and hypoxic stress typical of inflammation, contributes to the vessels formation, promoting angiogenesis and metastasis [101]. Blocking CXCR4 in the signal cascade leads to a reduction in tumor endothelium with a mechanism that is VEGF-independent [102]. Tumor-associated macrophages (TAMs) with the M2-like phenotype are related to tumor cell growth and spreading. In CRC, CXCL12/CXCR4 axis activation stimulates the up-regulation of miRNAs on TAMs, leading to enhanced immunosuppression, epithelial to mesenchymal transition (EMT), as well as angiogenesis via VEGF and EGF secretion [103]. The training/validation study by Matsusaka and colleagues, including a total of 874 Caucasian and Asian mCRC patients receiving first-line chemotherapy with bevacizumab, found that the *CXCR4*-rs2228014-CC genotype showed a longer PFS compared with CT/TT genotypes in multivariate analysis (training cohort, HR:1.77, *p* = 0.029; validation cohort B, HR:1.87, *p* = 0.009). These results remained significant after adjusting by primary tumor location for patients with left-sided mCRC [35].

TAMs-related functions are also regulated by several genes, including C-C motif chemokine ligand 2 (*CCL2*), C-C motif chemokine ligand 18 (*CCL18*), TANK-binding kinase 1 (*TBK1*), and IFN regulatory factor 3 (*IRF3*), whose signaling pathway is activated during tumor angiogenesis. In particular, TBK1 is triggered under hypoxic conditions and in turn activates IRF3 and NF-kB-dependent factors associated with inflammation [104]. TBK1 and IRF3 polymorphisms were investigated for their effects on tumor growth and vascularization and thus on response to bevacizumab therapy. The discovery-validation study by Sunakawa et al. on mCRC patients evidenced that carriers of the *TBK1*-rs7486100-T allele had shorter PFS than those with the AA genotype (HR:1.46, adjusted *p* = 0.028) in the wild-type *KRAS* validation set. Moreover, carriers of the *IRF3*-rs2304205-C allele experienced better RR compared to the AA genotype (79% vs. 54%, adjusted *p* = 0.0056) limited to the wild-type *KRAS* discovery cohort. The authors also highlighted that in the mutant *KRAS* discovery set, mCRC patients carrying *CCL2*-rs4586-C and *IRF3*-rs2304205-C alleles had longer PFS (HR:0.51, adjusted-*p* = 0.026, and HR:0.56, adjusted-*p* = 0.039 in the dominant model, respectively) compared to other genotypes in multivariable analysis. The *CCL18*-rs14304-TT genotype was associated with worse PFS compared to the CC genotype (HR:1.24, adjusted-*p* = 0.007) [36].

Reduced oxygen tension is crucial for tumor vascularization and regulates VEGF production. The modulation of both VEGF expression and tumor angiogenesis is also driven by molecules belonging to HIF1α and insulin-like growth factor 1 (IGF1)/insulin-like growth factor 1 receptor (IGF1R) signaling [105,106]. The role of polymorphisms in *IGF1R* was here reported because it was suggested that a decreased IGF1R expression may also affect CD4^+^ Th cell lineage commitment [107]. The response to bevacizumab-based therapy may be thus influenced by variants in *HIF1α*, *IGF1*, and *IGFR1* genes. A study conducted on 132 mCRC patients administered bevacizumab in association with fluoropyrimidines and oxaliplatin, evidenced that patients carrying the *HIF1a*-rs11549465-T allele had longer PFS (HR:0.55, *p* = 0.038) compared to the CC genotype in univariate analysis [28]. Moreover, carriers of a combination of *HIF1a*-rs11549465-CC, *VEGF*-rs699947-A, and *EGFR*-rs2227983-GG experienced shorter PFS (HR:2.66, *p* < 0.001). The *IGF1*-rs6220-G allele was associated with better overall survival (OS) compared to the AA genotype in multivariate analysis (HR:0.60, *p* = 0.046) [28]. In the FIRE 3 trial, 614 mCRC Caucasian patients receiving fluoropyrimidines and irinotecan-based chemotherapy plus bevacizumab or cetuximab were assessed for IGF and insulin receptor substrate (IRS) 1 and 2 isoforms. From multivariate analysis emerged that carriers of the *IGF1*-rs2946834-T allele had longer PFS (HR:0.78, *p* = 0.010) and OS (HR:0.65, *p* = 0.003) compared to carriers of the CC genotype. In the subgroup of FOLFIRI plus bevacizumab patients, *IGF1*-rs2946834-T allele carriers showed only a trend of longer PFS in the multivariable model (HR:0.77, *p* = 0.074) [38]. In the whole population analysis, the *IRS1*-rs1801123-C allele was correlated independent from *KRAS* status with shorter OS (HR:1.28, *p* = 0.054) than the TT genotype in multivariate analysis, resulting more a prognostic than a predictive biomarker of therapy response [38]. In 132 mCRC patients treated with first-line FOLFOX or XELOX and bevacizumab, the *IGF-1*-rs6220-G allele, associated with increased VEGF mRNA expression, showed increased OS (HR:0.51, *p* = 0.005) and remained significant in multivariate analysis (HR:0.60, adjusted-*p* = 0.046) [28]. Several studies have also suggested that the IGF1 pathway is a key mediator of resistance to cytotoxic chemotherapy and anti-EGFR treatment [108,109,110,111]. An analysis of 130 formalin-fixed, paraffin-embedded (FFPE) samples from patients with mCRC treated with a cetuximab-based regimen showed that polymorphisms in the IGF pathway were significantly associated with PFS and/or OS. In particular, *IGF1*-rs6214-TT was associated to worse PFS (HR:2.239, *p* = 0.008) and OS (HR:2.282, *p* = 0.011 in multivariate analysis), the *IGF1R*-rs2016347-G allele was associated with shorter OS (HR:1.734, *p* = 0.033 in multivariate analysis), while the *IGF1*-rs2946834-G allele was associated to shorter PFS (HR:2.943, *p* = 0.002 in multivariate analysis) [39].

Endothelial NOS (eNOS, also known as NOS3), constitutively expressed at the level of the endothelium, plays a key role in endothelial health during angiogenesis, as well as in the regulation of immune activation. *ENOS* is a gene involved in the production of nitric oxide (NO) and is therefore essential for hemodynamic maintenance of blood flow due to its ability to regulate blood vessel permeability. Production of NO by macrophages, dendritic cells, and NK increases levels of VEGF, which phosphorylates eNOS by binding to endothelial cells, stimulating cell production and thus the growth of new blood vessels [112]. Two immune pathways are involved in this process: HIF-1α induction and PGE2 production. HIF-1α is regulated by NO, but also by cytokines and growth factors. HIF-1α, as a cellular hypoxia sensor, induces genes that promote vasodilation, vascular permeability, and angiogenesis. Regulation of NO is also essential for tissue repair. Specifically, NO activates metalloproteinases (MMPs) that remodel the cellular matrix for neovascularization through proteolytic activity [112,113]. Variants in *ENOS* gene reduce the enzyme activity and/or expression and thus the NO levels may enhance the response to anti-angiogenic-based treatments. Ulivi et al. assessed 237 Caucasian mCRC patients and found that heterozygous carriers of *ENOS*-rs1799983-GT variant had shorter PFS (HR:1.70, *p* = 0.013), OS (HR:1.80, *p* = 0.014), and overall response rate (ORR) (42.5% vs. 63.1%, *p* = 0.030) compared to GG and TT carriers [40]. Moreover, patients with the *ENOS*-rs2070744-TT genotype had both shorter PFS (HR:3.63, *p* = 0.036) and OS (HR:5.48, *p* = 0.007) than CT/CC carriers. Another polymorphism reducing the protein expression is the intronic variable number tandem repeat (VNTR) of 27 nucleotides in the *ENOS* gene. Patients carrying *ENOS-VNTR-4bb* (homozygous for five repeats) showed both longer PFS (HR:0.63, *p* = 0.034) and OS (HR:0.54, *p* = 0.015) than patients carrying at least four repeats (*VNTR-4ba/4aa*). Combining haplotype (i.e., Haplo 1: *VNTR-b*/rs1799983-G and Haplo 2: *VNTR-b*/rs1799983-T) *ENOS* Haplo1/Haplo1 and Haplo2/Haplo2 showed an improved PFS (15.0 vs. 9.1 months, *p* = 0.001), OS (34.5 vs. 20.5 months, *p* = 0.002), and ORR (71% vs. 45.9%, *p* = 0.013) compared to other *ENOS* haplotype combinations [40]. *ENOS*-rs1799983 was investigated in 232 mutant *KRAS* Caucasian mCRC patients administered chemotherapy alone (n = 112) or chemotherapy plus bevacizumab (n = 120). In the bevacizumab group, patients carrying the *ENOS*-rs1799983-TT genotype, leading to a reduced NO production, had higher severe toxicity, particular grade 3-4 hypertension and proteinuria (GG+GT: 8% vs. TT: 50%; *p* = 0.0002) [41].

Another gene involved in the production of NO is *NOS2* coding for iNOS (also known as nitric-oxide synthase 2 [NOS2]), an NADPH-dependent enzyme known as a surrogate marker of M1 macrophage activation. In 227 mCRC patients receiving FOLFIRI plus bevacizumab, patients with CCTTT any >13 repeats showed improved median PFS compared with patients carrying the ≤13/≤13 repeats variant (HR:0.64, *p* = 0.010). Similar results were shown adopting the >26repeats/≤26 repeats (HR:0.56, *p* = 0.005). These data were partially confirmed in 231 patients receiving FOLFOXIRI plus bevacizumab: a better median PFS was observed in patients with >26 vs. ≤26 repeats. However, these data were not confirmed in the two validation cohorts of 301 and 187 patients [42]. These results seem to suggest that the *ENOS* gene has a more important role in maintaining endothelial cell functional integrity.

The reactive oxygen species (ROS) could be directly scavenged by different antioxidant molecules, including superoxide dismutase (SOD), catalase (CAT), glutathione peroxidase (GPx), and cytosolic and mitochondrial thioredoxins (TXN1 and TXN2). A study of 236 mCRC Caucasians, 129 patients treated with first-line FOLFIRI+cetuximab and 107 with FOLFIRI+bevacizumab, analyzed the effects of polymorphisms in genes involved in the antioxidant pathway [43]. In the FOLFIRI+cetuximab group, they found that the patients’ carrier *TXN2*-rs4821494-G allele and *GPX4*-rs4807542-A allele had worse OS, confirmed by multivariate analysis (HR:2.47, *p* = 0.03; HR:2.24, *p* = 0.05). The *TXN2*-rs4821494 polymorphism is located in the 5′UTR and the T mutant allele was related to a lower TXN2 expression in sigmoid and transverse colon tissue. The role of ROS is pleiotropic and it includes the inflammasome activation critical for an efficient immune response [114].

### 4.3. Nuclear Receptors

Nuclear receptors (NRs) are key sensors in regulating gene expression of proteins involved in drug transport and metabolism and in orchestrating the activation of the immune system triggered by xenobiotics themselves [11,12,115,116]. Particularly, the pregnane X receptor (PXR, *NR1I2*) is a major regulator of both xenobiotic detoxification and innate immunity to xenobiotics. PXR ligands stimulate the expression of NLR family pyrin domain containing 3 (NLRP3), nucleotide-binding oligomerization domain 1 (NOD1), and Toll-like receptor (TLR)-2, TLR-4, and TLR-9 in endothelial cells by causing activation of the inflammasome and simultaneous activation of PXR and innate immunity [117]. Activation of PXR, in turn, stimulates several phase 1 (e.g., cytochromes [CYPs]) and phase 2 enzymes (e.g., glutathione S-transferase [GSTs], sulfotransferase [SULTs], UDP-glucuronosyltransferase [UGT1As]), as well as the membrane ATP-binding cassette (ABC)/solute carrier (SLC) transporters that have a crucial role in the modulation of ADME of chemotherapeutics [11,12,115]. Overexpression of the PXR in vivo and in vitro models has been correlated with irinotecan resistance due to UGT1A induction [118]. By contrast, the active metabolite of irinotecan (SN-38) has been associated with PXR induction, improving irinotecan metabolism [119]. A study involving 109 advanced CRC patients receiving 180 mg/m^2^ irinotecan treatment highlighted a correlation between severe hematological toxicity and *NR1I2*-rs10934498-A (OR:0.17, *p* = 0.009), *NR1I2*-rs2472677-G (OR:41.55, *p* = 0.003), *NR1I2*-rs3814055-T (OR:9.25, *p* = 0.005), and *NR1I2*-rs1523127-C (OR:7.23, *p* = 0.009) alleles. In addition, *NR1I2*-rs2472677-G carriers experienced a higher grade of any type of toxicity than other genotypes (OR:6.78, *p* = 0.031) [44]. In a discovery/replication study involving about 400 mCRC patients treated with irinotecan-based therapy, those carrying the *NR1I2*-rs1054190-TT genotype had significant short OS (HR = 6.78, *p* = 0.0021, and HR = 3.56, *p* = 0.0414 in the discovery and replication sets, respectively) compared to carriers of the C ancestral allele [45].

The vitamin D receptor (VDR), another member of NRs, is a mediator of the genomic actions of 1,25-dihydroxycholecalciferol and, more recently, emerged as an immunomodulatory player. Polymorphisms of VDR and vitamin D3 (VitD3) serum levels have been correlated to (auto)immune-diseases development since VDR is expressed in several immune cell types [120]. The VitD3/VDR axis shows regulatory activity in the adaptive immune response, suggesting a possible influence of the nuclear receptor and its polymorphisms on tumor cell growth, inflammation, invasion, metastasis, and angiogenesis [121]. In addition, this protein regulates calcium and phosphate homeostasis as well as the transcription of genes affecting the metabolism of drugs (i.e., CYPs, UGT1As, ABC/SLC transporters) [45]. The study by De Mattia et al. highlighted an association of the *VDR*-rs7299460-T allele with longer OS (HR:0.61, *p* = 0.0076; HR:0.57, *p* = 0.0478 in discovery and replication sets, respectively). Additionally, a study involving 250 mCRC patients suggests that *VDR*-rs11574077 polymorphism is correlated with irinotecan-related toxicity. The pharmacokinetic analysis evidenced that the *VDR*-rs11574077-G minor allele affects the efficacy of irinotecan glucuronidation and detoxification. *VDR*-rs11574077-G carriers concordantly experienced higher grade gastrointestinal toxicity although limited to the discovery set (OR:4.46, *p* = 0.010) [29].

### 4.4. Toll-Like Receptors (TLRs)

The TME and immune responses not only play a key role in tumor growth and progression but could also influence the efficacy and toxicity of cancer therapies. TLRs, together with NOD-like receptors (NLRs), belong to pattern recognition receptors (PRRs) and can recognize a variety of pathogen-associated molecular patterns (PAMPs). In particular, TLRs expressed on numerous immune cells (e.g., macrophages, dendritic cells, NK cells, and T and B lymphocytes) and non-immune cells (e.g., endothelial cells, fibroblasts) are sensors of the innate immune response, and some members induce the production of type I interferons (IFN-α and IFN-β), which further stimulates the immune response [122]. TLRs are membrane-receptors located on the cell surface or endosomes with different specificities [123,124].

TLR-4, a bacterial sensor whose activation stimulates NF-κB signaling and inflammatory cytokine production, has been associated with the risk of developing gastrointestinal toxicity during irinotecan treatment, likely related to intestinal mucosal damage and associated infections [125]. In addition, irinotecan promotes activation of the innate immune response through direct binding to the TLR-4/MD-2 complex and stimulates the inflammatory response [126]. This mechanism appears to be confirmed in a preliminary, albeit small, study of patients with advanced CRC treated with irinotecan. Analysis showed that carriers of *TLR-4*-rs4986790-G and *TLR-4*-rs4986791-T minor alleles had increased IL-6 plasma levels and a more frequent occurrence of severe and late-onset diarrhea [49].

A study of 5000 patients with CRC, in which 40% had advanced disease, investigated the polymorphisms *FPR1*-rs867228, *TLR-3*-rs3775291, and *TLR-4*-rs4986790, and in other proteins of the PRRs family, without finding any significant association [48].

*TLR-7* polymorphisms have been investigated in cancer immunotherapy due to the ability of this PRR to induce a robust release of anti-cancer cytokines such as IL-12. A study by Okazaki et al. found *TLR-7*-rs3853839 to be a potential independent biomarker of PFS in patients treated with anti-EGFR therapy. Particularly, the multivariable model showed that patients carrying the *TLR-7*-rs3853839-GG genotype had longer PFS compared to those carrying GC/CC genotypes (HR:2.02, *p* = 0.015) [50].

As abovementioned, TLRs are also expressed on non-immune cells and, therefore, they also play a role in modulating the functions of endothelial cells via mitogen-activated protein kinase kinase kinase 7 (MAP3K7 or TAK1) signaling that controls cell viability and the inflammation response [127,128]. TLR-1 and TLR-6, both of which dimerize with TLR-2, play critical roles in the intestinal mucosal immune response. Genetic variants in these PRRs and their common downstream signaling molecule TAK1 could lead to interindividual differences in drug response in mCRC patients [129]. In a study of 535 mCRC patients treated with FOLFIRI and bevacizumab, patients with the *TLR-1*-rs5743618-TT genotype had a worse RR than GT/GG genotypes (discovery set: 43% vs. 62%, *p* = 0.025; validation set: 46% vs. 65%, *p* = 0.021). Patients with the *TLR-6*-rs5743818-AA genotype had a lower response rate (RR) and shorter PFS than AC/CC carriers, limited to univariate analysis. In the discovery arm, patients with the *TLR-1*-rs5743618-TT genotype had shorter PFS and OS compared with GT/TT genotypes (HR:1.50, *p* = 0.046; HR:1.53, *p* = 0.025, respectively). In the same group, patients with the *TAK1*-rs1145727-AA genotype had a shorter OS than patients with GA/GG genotypes (23 vs. 26.8 months, *p* = 0.008) [47]. The *TLR-1*-rs5743618 polymorphism is a missense (Ser602Ile) variation in which the presence of the mutant G allele is associated with a decrease in IL-6 and TNF-α levels and therefore with a reduction in TLR-1-mediated immunity [130]. This may explain the altered pro-tumorigenic activity linked to the worse response of patients carrying the *TLR-1*-rs5743618-T allele.

### 4.5. Cytokines and Chemokines in the Early Phase of Immune Response

Other pro-inflammatory cytokines, such as interleukin-8 (IL-8) and interleukin-12 (IL-12), play important roles as chemo-attractants for innate immune cells such as neutrophils, basophils, dendritic cells, eosinophils, Langerhans cells, mast cells, monocytes, macrophages, neutrophils, NK and T cells at the site of inflammation. IL-8, encoded by *CXCL8*, plays a role not only in altering vascular permeability by promoting neutrophil recruitment and migration but also in their activation, triggering the respiratory burst, thereby ROS and NO. Moreover, IL-8 has a pivotal role in the angiogenesis, proliferation, and migration of tumor cells as well as TME modulation [131,132]. Polymorphisms in *CXCL8* may have a role in the modulation of the abovementioned functions, possibly influencing the response to chemotherapy in the mCRC setting. Mutant RAS mCRC patients carrying the *CXCL8*-rs4073-TT genotype showed better PFS (HR:0.53, *p* = 0.002) and OS (HR:0.64, *p* = 0.03) after bevacizumab-based therapy compared to those carrying TA/AA genotypes but only PFS retained significance in multivariate analysis (HR:1.8, *p* = 0.0006). This interindividual variability in response to treatment was supported by different IL-8 serum levels: *CXCL8*-rs4073-TT genotype carriers had lower cytokine serum levels [41]. Another study involving 125 metastatic or relapsed CRC patients treated with first-line cytotoxic chemotherapy combined with bevacizumab correlated the *CXCL8*-rs4073-T allele to a tendency for longer PFS, whereas it was significantly associated with better ORR compared to the AA genotype (OR:0.394, *p* = 0.067) [51]. In another study with 108 *KRAS* wild-type patients treated with bevacizumab-based therapy, the presence of the *CXCL8*-rs4073-any A allele (HR:1.51, *p* = 0.037) and the *TBK1*-rs7486100-TT genotype (HR:1.94, *p* = 0.037) were associated with worse PFS [52]. The prognostic effect of the *CXCL8*-rs4073 variant is independent to the *KRAS* status but seems related to the interaction with bevacizumab-containing regimens. The role of this variant was also investigated in another three studies in patients with mCRC treated with cetuximab-based or oxaliplatin-based therapies or regorafenib; however, no significant associations with response, survival, and skin rash toxicity were found [31,53,54].

A study on 132 mCRC Caucasian patients administered first-line bevacizumab/oxaliplatin-based therapy investigated IL-8 receptors, CXCR1 and CXCR2. From the analysis emerged an association between the *CXCR1*-rs2234671-GG genotype and good RR (71% GG vs. 37% GC vs. 17% CC, *p* < 0.001). The *CXCR2*-rs2230054-CC genotype showed worse RR (62% TT vs. 44% TC vs. 21% CC, *p* = 0.008), retaining significance after multiple testing [28]. In Asian patients, the *CXCR2*-rs2230054 variant did not show any significant relation with bevacizumab-related efficacy outcome as well as the *IL10*-rs1800896, which was another biomarker with a role in the angiogenic pathway [51]. In 105 mCRC patients mainly Caucasians treated with 5-FU or oxaliplatin-based regimens without bevacizumab, the presence of *CXCR1*-rs2234671-GC compared to the GG genotype showed worse TTP while no association was reported for *CXCR2*-rs2230054 [53]. The *CXCR1*-rs2234671 was also included in a comprehensive pharmacogenetic profiling of biomarkers belonging to the EGFR pathway and its relation with response and toxicity in patients treated with cetuximab-based regimens was evaluated without finding significant association [33].

Finally, other pro-inflammatory cytokines, the IL-17A and IL-17F, are mainly produced by Th17 (T-helper cell type 17) cells in adaptive immunity, but they are also secreted by other immune cells including NK, NKT, neutrophils, and intestinal Paneth cells, highlighting their role also in the innate response [133]. Moreover, IL-17 was investigated for its involvement in several other pathways [134]. For example, a dysregulated IL-17A and IL-17F production can impair myeloid cell recruitment and determine an excessive pro-inflammatory cytokine expression, and consequently chronic inflammation, which leads to cancer development. A study in 122 Caucasians mCRC treated with bevacizumab-based therapy evaluated the predictive role of *IL17A*-rs2275913 and *IL17F*-rs763780 variants, however, no significant association with efficacy outcomes was found. Only the baseline serum IL-17A concentration was significantly associated with the response to bevacizumab [56].

### 4.6. Tumor Immune Escape Factors

Antigen presentation is a crucial step in the cell-mediated mechanisms essential for the activation of the immune system. Tumor cells could up-regulate the immune checkpoint and their ligands, inhibiting activity or inducing apoptosis of the immune cells (tumor immune escape mechanism). Programmed cell death ligand 1 (PD-L1, *CD274*), cytotoxic T lymphocyte-associated protein 4 (CTLA-4), Cluster of Differentiation 47 (CD47), and some other innate immune checkpoints have been investigated by Volz et al. in a total of 924 mCRC patients [57].

In the training cohort (cetuximab-based), the *CD274*-rs2297137-G allele was associated with worse tumor response (56% AA vs. 19% GA vs. 16% GG; *p* = 0.029), the *CTLA4*-rs231777-CT genotype with shorter PFS (multivariate HR: 1.76; *p* = 0.019), and the *CD24*-rs52812045-AA genotype with shorter median PFS and OS (multivariate PFS HR:3.18, *p* = 0.009; OS: HR:4.93, *p* = 0.001). The association of the *CD24*-rs52812045-AA genotype with shorter PFS was validated in 74 patients treated with oxaliplatin plus cetuximab (multivariate HR:2.12; *p* = 0.018). The *CD274*-rs2297137-A allele was also associated with prolonged OS in multivariate analysis in the additional control cohort 2 (FOLFIRI plus bevacizumab) (HR:0.65 *p* = 0.021) [57]. Moreover, in 141 mCRC Chinese patients treated with first-line bevacizumab-based regimens, the *CD274*-rs2297136-AA genotype was associated with better PFS (HR:1.68, *p* = 0.018) and OS (HR:1.88, *p* = 0.012) in the whole population and also in the *KRAS* mutant subgroup [58]. The binding of CD24, present on tumor cells, to Siglec-10, expressed on immune cells, causes inhibition of the immune response mediated by the tyrosine phosphatases SHP-1 and SHP-2. SHP-1/2 phosphorylate the immunoreceptor tyrosine-based inhibitory motif (ITIM) at the cytoplasmic end of Siglec-10, blocking TLR-mediated inflammation and immune response and promoting tumor cell escape from the immune system [135]. HIF1-α appears to modulate human leukocyte antigen-G (HLA-G) expression, a tolerogenic molecule proposed as an immune checkpoint [136,137], and the *HIF1A*-rs2244608 polymorphism has been associated with the efficacy of irinotecan-based CRC therapy. In particular, patients carrying the *HNF1A*-rs2244608-G allele had better PFS than patients with the AA genotype (HR:0.72, *p* = 0.002) [62]. HLA-G has been studied as an independent marker of efficacy in CRC [138,139,140], and its polymorphisms deserve further investigation in mCRC [140].

The regulation of the killing function of NK and T cells is mediated by a family of type I transmembrane glycoproteins called killer-cell immunoglobulin-like receptors (KIRs) that interact with MHC class I. NK cells are able to discriminate MHC class I in transformed and virus-infected cells, ignoring potential targets, expressing normal levels of autologous MHC class I, and leading to inhibition of antiviral and cytotoxic activities [141]. Genetic variants in the KIRs and their ligands, HLA class I (HLA-A, -B, -C), were investigated in 224 mCRC patients receiving FOLFIRI [58]. KIR3DL1/HLA-Bw4-I80 combination emerged as a positive predictive marker of survival (HR for T80:2.8, *p* < 0.001 and HR for no functional KIR/HLA interaction:1.8, *p* = 0.007) and the presence of the KIR2DL5A, 2DS5, 2DS1, 3DS1, and KIR3DS1/HLA-Bw4-I80 combination was associated with an increased rate of complete response (ORs ranging from 2.1 to 4.3) [58]. Most KIRs have an inhibitory effect on immune cells, while some, such as KIR2DS4, can activate immune cells. *KIR2DS4* encodes both a full-length protein (KIR2DS4f) and a truncated protein (KIR2DS4d) due to a deletion of 22 base pairs in exon 5. This truncated protein cannot attach to the cytoplasmic membrane and is therefore considered a non-functional receptor. In a small group of 70 mCRC with mutant *KRAS* and *FCGR2A*-His131 patients treated with cetuximab, a significantly longer OS was observed in patients with the *KIR2DS4d/4d* genotype compared with individuals carrying at least a functional receptor (KIR2DS4f) (HR:2.27, *p* = 0.026) [60].

Adenosine, which plays an immunosuppressive and angiogenic modulator role in the TME, is produced by the ectodinucleotidase enzymes, CD39 and CD73, which are expressed in the surface membrane of tumor cells, but also in B cells or regulatory T cells [142,143]. Bevacizumab-induced hypoxia stimulates the up-regulation of HIF1-α and consequently of CD39 and CD73 in cancer cells, leading to suppression of cytotoxic T lymphocytes (CTLs) and NK cells. Recently, a large study of 322 Caucasian patients with mCRC treated with FOLFIRI plus bevacizumab demonstrated that in the discovery cohort, patients with both *CD39*-s11188513-C allele and *CD73*-rs2229523 GG genotype had a shorter OS in multivariable analysis (HR:2.10, *p* = 0.031; HR:0.49, *p* = 0.026, respectively), and was also confirmed in the validation cohort (HR:1.53, *p* = 0.013; HR:0.62, *p* = 0.024, respectively) [61]. Interestingly, these results were not replicated in the control arm in which patients received cetuximab-based chemotherapy, suggesting that the adenosine pathway may be a promising marker of specific bevacizumab response [61]. Moreover, extracellular adenosine exerts its immunosuppressive effect on tumor cells via A2BR, one of its four receptors, promoting tumor growth and metastasis. Carriers of the *A2BR*-rs2015353-TT genotype had a favorable OS compared with patients with at least one minor C allele in the multivariable model (HR: 0.24, *p* = 0.004) [61].

### 4.7. Cytokines in the Late Phase of Immune Response

The main cytokines produced by T cells are known as interleukins (ILs). IL-15, a type I cytokine together with IL-2, IL-4, IL-7, IL-9, and IL-21, promotes survival of T, B, and NK cells by binding to IL-15α, encoded by *IL15RA*, negatively regulating both carcinogenesis and tumor growth [144]. IL-2, IL-4, IL-7, IL-9, and IL-15 receptors have a common gamma (γ_c_) chain, also referred to as CD132. Besides CD132, IL-15α shares a β chain (IL-2/15Rβ or CD122) with IL-2, a cytokine structurally related to IL-15. The binding of IL-15 to CD122 stimulates the phosphorylation of Jak3 and subsequent activation of STAT5. The Jak3/STAT5 pathway induces cell survival of T, NK, and NK-T lymphocytes and promotes the differentiation of NK lymphocytes by increasing the expression of anti-apoptotic proteins such as Bcl2 and Bcl-x [145]. The overproduction of soluble IL-15/IL-15Rα could represent a novel mechanism of immune escape [146]. Furthermore, IL-15, through the balance between IL-2 and IL-15, stimulates survival for CD8 memory T cells [147]. The main cytokine produced by the CD8 T cells is the IFNγ, which mainly increases the activation of MHC class I and II on macrophages. Variants in the INFG receptor gene (*INFGR1*) have been investigated for their predictive role in 141 Chinese mCRC treated patients with bevacizumab-based regimens. In particular, patients with the *IFNGR1*-rs2234711-G allele and *IFNGR1*-rs9376267-T allele showed longer OS only in univariate analysis (*p* = 0.041; *p* = 0.0312) [58].

The TGF-β/SMAD-3 pathway is involved in chemoresistance modulating the TME: TGF-β through SMAD3 inhibits CD4 T cell proliferation and effectors function, silencing the IL-2 expression [148]. In a recent study involving 335 mCRC patients who received first-line FOLFIRI, the *IL15RA*-rs7910212-C allele was associated with short OS (HR:1.57, *p* = 0.0327; HR:1.71, *p* = 0.0411 in discovery and replication sets, respectively) according to the dominant model. In the same analysis groups, the *SMAD3*-rs7179840-C allele was instead associated with longer OS (HR:0.65, *p* = 0.0202; HR:0.61, *p* = 0.0216 in discovery and replication sets, respectively) in the dominant model [46].

Other pathways involved in the immune system are C-C motif chemokine ligand 5 (CCL5)/C-C chemokine receptor type 5 (CCR5) axis, C-C motif chemokine ligand 3 (CCL3), and C-C motif chemokine ligand 4 (CCL4), which stimulate the dendritic cells to produce IL-12, induce migration of endothelial progenitor cells, leading to increased *VEGFA* expression. A study comprised two independent sets of mCRC patients (79 Asian and 150 Caucasian patients for discovery and validation set, respectively) treated with regorafenib as monotherapy [63], indicated that patients carrying the *CCL4*-rs1634517-A allele have significantly shorter PFS than those carrying the CC genotype (discovery set: HR:1.58, adjusted-*p* = 0.058; validation set: HR:1.59, adjusted-*p* = 0.012). In addition, patients carrying the *CCL3*-rs1130371-A allele were suggested to have a shorter PFS than those carrying GG (discovery set: HR:1.48, adjusted-*p* = 0.064; validation set: HR:1.50, adjusted-*p* = 0.027). The *CCL4*-rs1634517-A and *CCL3*-rs1130371-A alleles resulted also in predictors of shorter OS in the validation set (HR:1.46, adjusted-*p* = 0.041, and HR:1.44, adjusted-*p* = 0.047, respectively) [63]. Interestingly, in a later analysis of the same cohorts, Suenaga et al. correlated polymorphisms in the CCL5/CCR5 pathway with serum levels of the CCR5 circulating protein and its ligands. From the study emerged that patients carrying the *CCL5*-rs2280789-G allele had higher CCL3 but lower CCL4 serum levels compared to AA carriers (72.7% vs. 23.1%, *p* = 0.006 and 31.8% vs. 69.2%, *p* = 0.043, respectively) [65]. Considering that the levels of these markers (increased CCL3 and decreased CCL4) are related to a good response to regorafenib, their association with *CCL5*-rs2280789 polymorphism suggests a mechanism of action in the CCR5 network beyond the CCL5/VEGFA pathway.

The role of polymorphism in two enterocyte-specific genes, *MS4AI2* and its transcriptional activator *CDX2*, in predicting the efficacy of oxaliplatin was also investigated in a total of 604 mCRC patients who were divided into a discovery cohort (146, FOLFOX +/− bevacizumab), a validation cohort (230, FOLFOXIRI plus bevacizumab), and a control cohort (228, FOLFIRI plus bevacizumab) [67]. Enterocytes play a role in the absorption of small molecules from the lumen and in intestinal immunity by secreting antimicrobial proteins and cytokines and acting as antigen-presenting cells (APCs) for intestinal T lymphocytes. The *MS4A12*-rs4939378-G allele was correlated to longer PFS than the AA genotype in multivariate analysis (HR:0.65, *p* = 0.035) in patients expressing the wild-type *KRAS*. Only in the mutant *KRAS* subgroup, patients with the *CDX2*-rs3812863-GG variant showed longer PFS than those with any A allele in univariate analysis (HR:0.39, *p* = 0.004), pointing out the prognostic role of these biomarkers [67].

### 4.8. Regulated Cell Death Factors

Regulated cell death is a form of cell death due to signal transduction that can be pharmacologically or genetically modulated. The cytotoxic T cells induce the apoptosis of their targets both through the release of cytotoxic granules and also contribute to the immune homeostasis maintenance through the Fas/FasL interaction that leads to activation-induced cell death. This last form of apoptosis is induced by repeated T cell receptor (TCR) stimulation, responsible for the peripheral deletion of activated T cells through the caspases [149].

Alterations in genes involved in homeostasis maintenance through the apoptosis mechanism are investigated in mCRC. However, in 76 mCRC patients treated with XELOX chemotherapy, no association with response and survival were found for polymorphisms in *Caspase 3*, *6-10*, *TP53*, *BCL2L*, *TNFRSF10B*, *AKT1*, *BID*, *RIPK1*, *FAS*, and *FASL* genes [32]. Moreover, the autophagy-related gene *ATG2B* was investigated for its prognostic role in 325 Chinese patients treated with oxaliplatin-based or irinotecan-based chemotherapy. The *ATG2B*-rs17094017-T allele was associated with an increased OS (HR:0.65, *p* = 0.002), PFS (HR:0.76, *p* = 0.007), and DCR (OR:0.60, *p* = 0.03) in the overall population, though stratifying by the chemotherapy used, these effects were confirmed only for patients treated with oxaliplatin-based regimen (OS, HR:0.64, *p* = 0.0219; PFS, HR:0.72, *p* = 0.0215; DCR, OR:0.39, *p* = 0.126) [88]. Further investigation should be done to better identify the functional role of this polymorphism on the ATG2B expression and the exact mechanism underlying its influence on the immune system.

Some chemotherapeutic agents, including oxaliplatin, are capable of triggering a process known as immunogenic cell death (ICD) [150]. Oxaliplatin-based treatments lead to the production of DAMPs in ICD by stimulating the release of calreticulin (CALR), annexin A1 (ANXA1), and high-mobility group box 1 (HMGB1). These factors are recognized by PRRs, including LDL protein-related receptor 1 (LRP1) and purinergic receptor P2X 7 (P2RX7) [150]. In a study of 648 mCRC patients with discovery/validation design treated with an oxaliplatin-containing regimen (161 FOLFOX/bevacizumab in the discovery cohort, 109 FOLFOXIRI/bevacizumab in validation cohort; and 378 FOLFIRI/bevacizumab in two control cohorts), both the *ANXA1*-rs1050305-AA and *LRP1*-rs1799986-CC genotypes correlated with better OS [87]. Particularly, the *ANXA1*-rs1050305-AA genotype showed longer OS compared to any G allele both in the discovery and validation cohorts (HR:1.87, *p* = 0.03; HR:2.69, *p* < 0.001, respectively) and *LRP1*-rs1799986-CC had better OS than any T allele (HR:1.69, *p* = 0.03) although not confirmed in the validation cohort [87]. In the discovery cohort, the *CALR*-rs1010222-A allele had a better PFS in univariate analysis (HR:0.61, *p* = 0.008) compared to the GG genotype, with only a concordant trend in multivariate analysis. This association was not confirmed in the validation cohort.

Destruction of tumor cells mediated by the binding of the antibodies (including cetuximab) through the crystalline fragment (Fc) receptors of NK cells is termed ADCC [18,20]. ADCC represents a mechanism by which an effector cell without antigenic specificity can mediate antigen-specific functions by recognizing the Fc portions of antibodies used to bind to the tumor cell. Activated NKs lyse tumor cells and stimulates the immune response via cross-talk with dendritic cells (DCs), and macrophages, via IFNγ, chemokines, and cytokines. Tumor cell lyses, by lytic granules, further stimulate the release of tumor antigens, which triggers additional cytotoxic activity via presentation to cytotoxic T cells by DCs [20,83]. The FcγRs are encoded by *FCGR1 (CD64)*, *FCGR2 (CD32)*, and *FCGR3 (CD16)* in chromosome 1, and polymorphisms in the Fc receptor of immune cells, altering the affinity for IgG Fc fragments, may influence the response to cetuximab proving to be a promising host genetic biomarker. Currently, a couple of SNPs have been associated with different IgG affinities: *FCGR2A*-rs1801274 (535 A > G, Arg131His) and *FCGR3A*-rs396991 (818 A > C, Val158Phe). The *FCGR2A*-rs1801274-His131 allotype displays a greater affinity for IgG1 compared to Arg131, whereas the *FCGR3A*-rs396991-Val158 allotype showed an increased affinity for IgG1, IgG2, and IgG3 compared to Phe158, improving the immune response through ADCC activation [151,152]. However, investigations on *FCGR* polymorphisms in mCRC patients are not yet conclusive as to their hypothetical significance at the clinical level. Despite the correlation between these polymorphisms and IgG binding affinity, some studies in mCRC patients showed no significant results assessing *FCGR2A*-rs1801274 and/or *FCGR3A*-rs396991 genotypes and cetuximab efficacy in terms of PFS, OS, and RR, irrespective of KRAS status [31,60,68,69,70,71,83]. However, in the study by Geva et al., an exploratory analysis in the subgroup of mutant *KRAS* mCRC patients, *FCGR3A*-rs396991-Phe/Phe was correlated to increase disease control rate (DCR) and OS compared to other genotypes (Pearson χ2 *p* = 0.042 Phe/Phe vs. non-Phe/Phe and Log-Rank *p* = 0.030 Phe/Phe vs. non-Phe/Phe, respectively) [68]. In the NCIC CTG CO.17 trial, the subgroup of wild-type *KRAS* patients carrying *FCGR2A*-rs1801274-His/His had both improved OS and PFS, demonstrating respectively a 5.5 months benefit (HR:0.36, *p* = 0.003) and a 3.7 months benefit (HR:0.19, *p* = 0.02) compared to carriers of the Arg allele [80]. Shepshelovich and colleagues highlighted similar results by assessing 572 Australian wild-types *KRAS* mCRC patients. Those harboring *FCGR2A*-rs1801274-His/His showed longer OS (HR:0.66, *p* < 0.001) compared to any Arg. Despite *FCGR3A*-rs396991 genotypes not being associated with clinical outcomes, an advantage in terms of OS was found combining *FCGR2A*-rs1801274-His/His and *FCGR3A*-rs396991-PhePhe (HR:0.33, *p* = 0.003) compared to *FCGR2A*-rs1801274-Arg/Arg and *FCGR3A*-rs396991-Val/Val [81]. The role of *FCGR3A*-rs396991 polymorphism was evaluated in the meta-analysis by Ying and colleagues including 16 trials and a total of 2831 mCRC patients administered cetuximab-based chemotherapy. The analysis evidenced an improved PFS for patients carrying *FCGR3A*-rs366991-Phe/Phe in the dominant model considering both the overall population (MSR:0.680, *p* = 0.027) and the wild-type *KRAS* subgroup (MSR:0.728, *p* = 0.12) [72]. A study involving 96 mCRC Caucasian patients highlighted a significant correlation between the *FCGR3A*-rs366991-Val allele and longer PFS compared to Phe/Phe (10.8 vs. 5.1 months respectively, *p* = 0.05) [73]. Contrasting results were evidenced in a previous small study in which mCRC patients carrying *FCGR3A*-rs396991-Val/Val had shorter PFS (Phe/Phe: 2.3 vs. Val/Phe: 2.4 vs. Val/Val: 1.1 months; *p* = 0.055). However, the combination of *FCGR2A*-rs1801274-Arg/Arg and *FCGR3A*-rs396991-Val/Val highlighted a shorter median PFS compared to other combinations (1.1 vs. 3.7 months, *p* = 0.001) [74]. A later combination analysis evidenced that mCRC patients carrying *FCGR2A*-rs1801274-His/His and/or *FCGR3A*-rs396991-Val/Val genotypes had longer PFS than carriers of Arg and Phe, respectively (5.5 vs. 3.0 months; *p* = 0.005) [75]. Other latest studies of comparable size attempted to define the role of *FCGR3A*-rs1396991 and *FCGR2A*-rs1801274 polymorphisms about cetuximab efficacy generating, however, contrasting results [76,77,78,82,86]. Because the main toxicity of cetuximab is skin rash and the high degree of toxicity has very often been correlated with a good response to the drug, some studies have attempted to evaluate these polymorphisms as markers of toxicity, but insignificant results have been obtained [31,70,78]. Overall, *FCGR2A* and *FCGR3A* polymorphisms emerge as promising biomarkers for predicting the efficacy of cetuximab. However, further prospective studies are needed to conclusively define the role of the rs1801274 and rs396991 polymorphisms concerning cetuximab efficacy, including consideration of *KRAS* status.

## 5. Conclusions

In recent years, the growing number of studies on metastatic colorectal cancer has highlighted how the different mechanisms of tumor onset, as well as the pathways and mechanisms of evasion of cancer cells, are strictly interconnected with the immune system, making further investigations necessary. At present, the inflammation and immune-related germline genetic markers emerge as promising predictors of the therapy outcome in mCRC patients, representing a further potential tool for personalizing and optimizing treatments. Most of the available data regard the impact of these markers on the therapy effectiveness while their effect on the risk to develop severe toxicity remains limited (Table 2).

**Figure 3:**
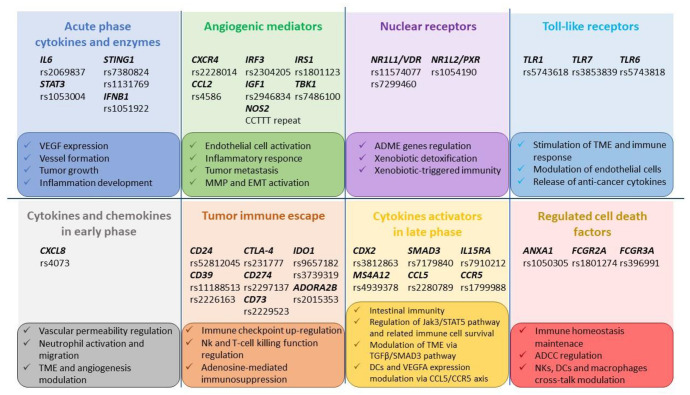
Promising genes and related pathways influenced by polymorphisms. **Abbreviations**: VEGF: Vascular Endothelial Growth Factor; MMP: Matrix metalloproteinases; EMT: Epithelial-mesenchymal transition; ADME: absorption, distribution, metabolism, and excretion; TME: tumor microenvironment; Nk: Natural Killer; DCs: Dendritic Cells.

Several studies focused on the impact of polymorphisms in genes belonging to ADCC (*FCGR2A*, *FCGR3A*) and yielded promising, but not conclusive, results on cetuximab efficacy. The remaining published data are sparse and have mainly hypothesis-generating value, suggesting, however, potentially interesting topics for future pharmacogenetic studies. Particularly, in addition to the tumor immune escape pathway, genetic markers belonging to the cytokines/interleukins, including *CXCL8*, *CCL5*, *CCR5* and angiogenic mediators including *IGF1*, *IRF3*, *NOS2*, appear to be the most promising (Figure 3).

The data published to date on the predictive role of genetic variants of the acute phase, innate, and adaptive immune responses may also lead to a better understanding of the involvement of immune regulation in modulating treatment outcomes and mechanisms of treatment resistance. In particular, genetic variants belonging to angiogenic mediators (*CXCR4*, *CCL2*, *CXCL8*, *TBK1*, *IRF3*) and tumor immune escape molecules (*CD24*, *CTLA-4*, *CD274*, *IDO1*) are mainly investigated in anti-VEGF and anti-EGFR therapies, respectively, generating promising, although preliminary data, suggesting a potential translation, after validation, into clinical practice. Furthermore, these data may provide information regarding possible novel biological targets for putative immunotherapies and vaccine strategies. Finally, the discovery of new immune/pharmacogenetics markers could ameliorate the current CMS classification in CRC, identifying further cancer subtypes that may be helpful in further personalizing treatments. In fact, alteration in genes with an immunosuppressive role could compromise the immune system homeostasis, causing an increase in its activity and a prolonged state of chronic inflammation, and then favoring tumor progression and therapy ineffectiveness.

In addition to the host genetic variants, other inflammation and immune-related molecular markers have been indicated to be helpful in predicting the outcome of mCRC treatment. Somatic biomarkers, such as Immunoscore, immunoprofiling, or tumor mutational burden (TMB), have been proposed to have a predictive or prognostic value [153,154], although their implementation in clinical practice is still tricky, mainly for the limited availability of tumor tissues. However, noninvasive approaches based on the body fluids of patients could overcome this limit. In particular, liquid biopsy approaches, such as noninvasive genetic tests, isolating tumor-derived entities could monitor the tumor and its development by an analysis of immune-related genomic and proteomic data [155]. In addition, both the germline variant profile of patients and immune system biomarkers monitored during chemotherapy can help predict survival, response to treatment, or development of toxicity.

Last but certainly not least, the increasing knowledge about the composition of the gut microbiota has made the interaction between the microbiota and the immune system a new field of interest. The composition of the microbiota, polymorphisms that alter immune-related genes, as well as genes involved in the production of reactive oxygen species and reactive nitrogen species, could adversely affect immune homeostasis, which is a key factor in preventing the development of cancer and influencing the effects of pharmacological treatments.

In conclusion, according to the literature data discussed in this review, the study of inflammatory and immune-related genetic markers is certainly an important and essential topic that should be the goal of future studies aimed at personalizing the treatment of mCRC.

## Figures and Tables

**Table 1:** Summary of the efficacy and toxicity evidence for immune-related genes divided in 8 groups belonging to the acute-phase/innate and adaptive immune response.

Genes	rs Code/ Alias	Therapy (N Patients in Cohorts)	Patients	Ethnicity	Clinical Outcomes	Level of Evidence	Main Effect	Ref.
**Acute-phase cytokines and enzymes**								
*IL6*	rs2069837	FOLFIRI+BV (223 training, 228 validation), FOLFIRI+CTX (264 control)	775	Caucasian	Efficacy	Moderate	rs2069837-G allele: lower mPFS (training, validation). rs2069837-AA genotype: higher tumor response (training, validation). No significant association with OS.	[27]
	rs1800795	FOLFIRI+BV (223 training, 228 validation), FOLFIRI+CTX (264 control)	775	Caucasian	Efficacy	Moderate	No significant association with RR, PFS, OS.	[27]
		BV+FOLFOX/XELOX	132	Caucasian	Efficacy	Low	rs1800795-G allele: higher OS.	[28]
*STAT3*	rs744166; rs4796793	FOLFIRI+BV (223 training, 228 validation), FOLFIRI+CTX (264 control)	775	Caucasian	Efficacy	Moderate	No significant association with RR, PFS, OS.	[27]
	rs1053004	FOLFIRI (250 discovery, 167 validation)	417	Caucasian	Toxicity	Moderate	rs1053004-C allele: lower risk of grade 3–4 GI toxicity.	[29]
*IL1RN* (IL-1RA)	rs4251961; rs579543	FOLFOX/FOLFIRI	180	Not found	Efficacy	Low	rs4251961-TT or rs579543-TT genotypes: higher survival.	[30]
*PTGS2* (COX-2)	rs5275	CTX	130	Mainly Caucasian	Efficacy/ Toxicity	Low	rs5275-T allele: better PFS. No associations with OS, ORR, skin rash toxicity.	[31]
		XELOX	76	Asian	Efficacy	Very low	rs5275-TT genotype: better PFS and OS. No significant association with ORR, skin rash toxicity.	[32]
		mFOLFOX/XELOX ± CTX	815	Caucasian	Efficacy/ Toxicity	Moderate	No significant association with RR, skin rash toxicity.	[33]
	rs20417	CTX	130	Mainly Caucasian	Efficacy/ Toxicity	Low	rs20417-GG: shorter mPFS. No significant association with RR, skin rash toxicity.	[31]
*STING1* (STING)	rs7380824	FOLFIRI+CTX (129 CTX cohort), FOLFIRI+BV (107 control cohort 1, 215 control cohort 2)	451	Caucasian	Efficacy	Moderate	rs7380824-any T allele: lower ORR in CTX cohort.	[34]
	rs1131769	FOLFIRI+CTX (129 CTX cohort), FOLFIRI+BV (107 control cohort 1, 215 control cohort 2)	451	Caucasian	Efficacy	Moderate	rs1131769-any T allele: shorter OS in CTX cohort.	[34]
*CGAS* (cCAS)	rs610913; rs311678; rs6907936	FOLFIRI+CTX (129 CTX cohort), FOLFIRI+BV (107 control cohort 1, 215 control cohort 2)	451	Caucasian	Efficacy	Moderate	No significant association with ORR, PFS, OS.	[34]
*IFNB1*	rs1051922	FOLFIRI+CTX (129 CTX cohort), FOLFIRI+BV (107 control cohort 1, 215 control cohort 2)	451	Caucasian	Efficacy	Moderate	rs1051922-G/A and A/A genotype: shorter PFS in CTX cohort.	[34]
	rs10964831	FOLFIRI+CTX (129 CTX cohort), FOLFIRI+BV (107 control cohort 1, 215 control cohort 2)	451	Caucasian	Efficacy	Moderate	No significant association with ORR, PFS, OS.	[34]
**Angiogenic mediators**								
*CXCR4*	rs2228014	FOLFOX/XELOX+BV (144 training), FOLFIRI+BV (424 validation A), FOLFOXIRI+BV (229 validation B), FOLFOX/SOX+CTX (77 control)	874	Asian and Caucasian	Efficacy	Moderate	rs2228014-T allele: shorter mPFS and trend of shorter mOS.	[35]
*CXCL12*	rs1801157; rs3740085	FOLFOX/XELOX+BV (144 training), FOLFIRI+BV (424 validation A), FOLFOXIRI+BV (229 validation B), FOLFOX/SOX+CTX (77 control)	874	Asian and Caucasian	Efficacy	Moderate	No significant association with PFS, OS, ORR.	[35]
*CCL2*	rs4586	FOLFIRI+BV (228 discovery, 248 validation), FOLFIRI+CTX (248 control)	724	Caucasian	Efficacy	Moderate	rs4586-C allele: better PFS in KRAS mutant of discovery cohort. No significant association with OS, RR.	[36]
*CCR2*	rs3092964	FOLFIRI+BV (228 discovery, 248 validation), FOLFIRI+CTX (248 control)	724	Caucasian	Efficacy	Moderate	No significant association with PFS, OS, ORR.	[36]
*CCL18*	rs14304	FOLFIRI+BV (228 discovery, 248 validation), FOLFIRI+CTX (248 control)	724	Caucasian	Efficacy	Moderate	rs14304-T allele: longer PFS (not validated in FIRE-BV cohort). No significant association with OS, RR.	[36]
*TBK1*	rs7486100; rs12313449	FOLFIRI+BV (228 discovery, 248 validation), FOLFIRI+CTX (248 control); FOLFIRI+BV (486)	486–724	Caucasian	Efficacy	Moderate	rs7486100-T allele: worse PFS in BV cohorts, worse OS only in KRAS wild-type. No significant association with OS, RR in all population.	[36,37]
*IRF3*	rs2304205; rs10415576	FOLFIRI+BV (228 discovery, 248 validation), FOLFIRI+CTX (248 control)	724	Caucasian	Efficacy	Moderate	rs2304205-C allele: better PFS in KRAS mutant of discovery cohort. No significant association with OS, RR.	[36]
*IGF1*	rs6220	BV+FOLFOX/XELOX	132	Caucasian	Efficacy	Low	rs6220-G allele: increased OS. No significant association with RR and PFS.	[28]
		FOLFIRI+BV (295), FOLFIRI+CTX (305)	614	Caucasian	Efficacy	Moderate	No significant association with PFS, OS.	[38]
		CTX	130	Caucasian	Efficacy	Low	No significant association with ORR, PFS, OS.	[39]
	rs6214	CTX	130	Caucasian	Efficacy	Low	rs6214-TT genotype: worse PFS and OS.	[39]
		FOLFIRI+BV (295), FOLFIRI+CTX (305)	614	Caucasian	Efficacy	Moderate	No significant association with PFS, OS.	[38]
	rs2946834	CTX	130	Caucasian	Efficacy	Low	rs2946834-AA genotype: better PFS in all population and better PFS, ORR in RAS wild-type subgroup.	[39]
		FOLFIRI+BV (295), FOLFIRI+CTX (305)	614	Caucasian	Efficacy	Moderate	rs2946834-A allele: better PFS in all population and better PFS, OS in RAS wild-type subgroup.	[38]
	rs7136446	CTX	130	Caucasian	Efficacy	Low	rs7136446-A allele: worse PFS in all patients. No significant association with OS, ORR.	[39]
*IGF1R*	rs2016347	CTX	130	Caucasian	Efficacy	Low	rs2016347-G allele: worse OS in all population and in wild-type KRAS subgroup. No significant association with PFS, ORR.	[39]
		FOLFIRI+BV (295), FOLFIRI+CTX (305)	614	Caucasian	Efficacy	Moderate	No significant association with PFS, OS.	[38]
	rs2272037	CTX	130	Caucasian	Efficacy	Low	rs2272037-C allele: worse OS. No significant association with ORR, PFS.	[39]
	rs2229765	CTX	130	Caucasian	Efficacy	Low	No significant association with ORR, PFS, OS.	[39]
*IRS1*	rs1801123	FOLFIRI+BV (295), FOLFIRI+CTX (305)	614	Caucasian	Efficacy	Moderate	rs1801123-C allele: worse OS in all population and in RAS wild-type. No significant association with PFS.	[38]
	rs1801278	FOLFIRI+BV (295), FOLFIRI+CTX (305)	614	Caucasian	Efficacy	Moderate	No significant association with PFS, OS.	[38]
*IRS2*	rs2289046; rs1805097	FOLFIRI+BV (295), FOLFIRI+CTX (305)	614	Caucasian	Efficacy	Moderate	No significant association with PFS, OS.	[38]
*NOS3/ENOS* (eNOS)	rs2070744	BV+FOLFOX/FOLFIRI (114 study), FOLFOX/FOLFIRI (123 control)	237	Caucasian	Efficacy	Low	*ENOS* Haplo1/Haplo1: longer mPFS; combining *ENOS* Haplo1/Haplo1 and *ENOS* Haplo 2/Haplo 2: longer PFS, OS. No significant association with RR.	[40]
		BV+FOLFOX6 (120 study), FOLFOX6 (112 control)	232	Caucasian	Efficacy/ Toxicity	Low	No significant association with ORR, PFS, OS, toxicity.	[41]
	rs1799983	BV+FOLFOX/FOLFIRI (114 study), FOLFOX/FOLFIRI (123 control)	237	Caucasian	Efficacy	Low	rs1799983-GT: worse ORR, PFS, OS. *ENOS* Haplo1/Haplo1: longer mPFS; combining *ENOS* Haplo1/Haplo1 and *ENOS* Haplo 2/Haplo 2: longer PFS, OS.	[40]
		BV+FOLFOX6 (120 study), FOLFOX6 (112 control)	232	Caucasian	Efficacy/ Toxicity	Low	rs1799983-TT genotype: higher severe toxicity in BV-based group. No significant association with ORR, PFS, OS.	[41]
	VNTR 4a/b 27pb	BV+FOLFOX/FOLFIRI (114 study), FOLFOX/FOLFIRI (123 control)	237	Caucasian	Efficacy	Low	VNTR-4bb: longer PFS, OS. *ENOS* Haplo1/Haplo1: longer mPFS; combining *ENOS* Haplo1/Haplo1 and *ENOS* Haplo 2/Haplo 2: longer PFS, OS. No significant association with RR.	[40]
*NOS2/INOS * (iNOS)	rs27779248	BV+FOLFIRI/FOLFOXIRI (227+231 evaluation cohorts), BV+FOLFIRI/FOLFOXIRI (301+187 validation cohorts)	946	Caucasian	Efficacy	Moderate	No significant association with PFS, OS, RR.	[42]
	CCTTT repeat	BV+FOLFIRI/FOLFOXIRI (227+231 evaluation cohorts), BV+FOLFIRI/FOLFOXIRI (301+187 validation cohorts)	946	Caucasian	Efficacy	Moderate	CCTTT >13repeats variant: better mPFS in BV+FOLFIRI. CCTTT >26 repeats variant: better mPFS in BV+FOLFIRI, partially confirmed in BV+FOLFOXIRI (not validated).	[42]
*HIF1A*	rs11549465	BV+FOLFOX/XELOX	132	Caucasian	Efficacy	Low	rs11549465-T allele: increased PFS only in univariate analysis. However, in the construction of the decision tree was the most important factor that determines PFS.	[28]
*TXN*	rs2301242	FOLFIRI+CTX (129 CTX cohort), FOLFIRI+BV (107 BV cohort)	236	Caucasian	Efficacy	Low	No significant association with PFS, tumor response neither in FOLFIRI+CTX or in FOLFIRI+BV.	[43]
*TXN2*	rs4821494	FOLFIRI+CTX (129 CTX cohort), FOLFIRI+BV (107 BV cohort)	236	Caucasian	Efficacy	Low	rs4821494-any G allele: worse OS in FOLFIRI+CTX. No significant association with PFS, tumor response in FOLFIRI+CTX, neither in FOLFIRI+BV.	[43]
	rs9619611; rs59841625	FOLFIRI+CTX (129 CTX cohort), FOLFIRI+BV (107 BV cohort)	236	Caucasian	Efficacy	Low	No significant association with PFS, tumor response neither in FOLFIRI+CTX or in FOLFIRI+BV.	[43]
*CAT*	rs7943316; rs564250; rs11604331; rs1001179; rs769217	FOLFIRI+CTX (129 CTX cohort), FOLFIRI+BV (107 BV cohort)	236	Caucasian	Efficacy	Low	No significant association with PFS, tumor response neither in FOLFIRI+CTX or in FOLFIRI+BV.	[43]
*GPX4*	rs4807542	FOLFIRI+CTX (129 CTX cohort), FOLFIRI+BV (107 BV cohort)	236	Caucasian	Efficacy	Low	rs4807542-any A allele: worse OS in FOLFIRI+CTX. No significant association in FOLFIRI+BV.	[43]
	rs757229; rs713041	FOLFIRI+CTX (129 CTX cohort), FOLFIRI+BV (107 BV cohort)	236	Caucasian	Efficacy	Low	No significant association with PFS, tumor response neither in FOLFIRI+CTX or in FOLFIRI+BV.	[43]
**Nuclear receptors**								
*NR1I2* (PXR)	rs10934498	FOLFIRI/FOLFIRINOX ± BV/CTX	109	Caucasian (French)	Toxicity	Low	rs10934498-A allele: decrease risk of grade 3-4 hematotoxicity.	[44]
	rs1523127	FOLFIRI/FOLFIRINOX ± BV/CTX	109	Caucasian (French)	Toxicity	Low	rs1523127-C allele: increased risk of grade 3–4 hematotoxicity.	[44]
	rs2472677	FOLFIRI/FOLFIRINOX ± BV/CTX	109	Caucasian (French)	Toxicity	Low	rs2472677-G allele: increased risk of all type of grade 3–4 toxicity and of grade 3–4 hematotoxicity.	[44]
	rs3814055	FOLFIRI/FOLFIRINOX ± BV/CTX	109	Caucasian (French)	Toxicity	Low	rs3814055-T allele: increased risk of grade 3–4 hematotoxicity.	[44]
	rs1054190	FOLFIRI (247 Italian discovery, 90 Canadian replication); FOLFIRI (250 Italian discovery, 92 Canadian replication)	337; 335	Caucasian	Efficacy	Moderate	rs1054190-TT genotype: short OS. Highly predictive genetic score when combined with *IL15RA*-rs7910212-TC/CC, *SMAD3*-rs7179840-TT, *VDR*-rs7299460-CC.	[45,46]
*NR1I3* (CAR)	rs2307418; rs2307424; rs2501873; rs2502815; rs3003596; rs4073054; rs6686001	FOLFIRI/FOLFIRINOX ± BV/CTX	109	Caucasian (French)	Toxicity	Low	No significant association with toxicity.	[44]
*NR1I1* (VDR)	rs7299460	FOLFIRI (247 Italian discovery, 90 Canadian replication); FOLFIRI (250 Italian discovery, 92 Canadian replication)	337; 335	Caucasian	Efficacy	Moderate	rs7299460-T: longer OS. Highly predictive genetic score when combined with *IL15RA*-rs7910212-TC/CC, *SMAD3*-rs7179840-TT, *NR1I2*-rs1054190-TT, *VDR*-rs7299460-CC.	[45,46]
	rs11574077	FOLFIRI	250	Caucasian	Toxicity	Moderate	rs11574077-G carriers: higher grade gastrointestinal toxicity limited to discovery set.	[29]
**Toll-like receptors**								
*TLR1*	rs5743618	FOLFIRI+BV (228 discovery, 297 validation)	525	Caucasian	Efficacy	Moderate	rs5743618-TT genotype: worse RR (validated), worse PFS and OS only in the discovery cohort (not validated).	[47]
	rs5743565	FOLFIRI+BV (228 discovery, 297 validation)	525	Caucasian	Efficacy	Moderate	No significant association with RR, PFS, OS.	[47]
*TLR2*	rs3804099	FOLFIRI+BV (228 discovery, 297 validation)	525	Caucasian	Efficacy	Moderate	rs3804099-C allele: better PFS only in univariate analyses (not validated). No significant association with RR, OS.	[47]
	rs4696480	FOLFIRI+BV (228 discovery, 297 validation)	525	Caucasian	Efficacy	Moderate	rs4696480-T allele: worse PFS only in univariate analyses (not validated). No significant association with RR, OS.	[47]
*TLR3*	rs3775291	CTX+OXA-based	1948	Caucasian	Efficacy	Moderate	No significant association with DFS, OS.	[48]
*TLR4*	rs4986790	IRI/FOLFIRI	46	Brazilian	Toxicity	Very low	rs4986790-(AG+GG) genotypes: more likely to experience diarrhea.	[49]
	rs4986790	CTX+OXA-based	1948	Caucasian	Efficacy	Moderate	No significant association with DFS or OS.	[48]
	rs4986791	IRI/FOLFIRI	46	Brazilians	Toxicity	Very low	TLR4-rs4986791-(CT+TT) genotypes: more likely to experience diarrhea.	[49]
	rs4986791	CTX+OXA-based	1948	Caucasian	Efficacy	Moderate	No significant association with DFS, OS.	[48]
*TLR6*	rs3821985	FOLFIRI+BV (228 discovery, 297 validation)	525	Caucasian	Efficacy	Moderate	No significant association with ORR, PFS, OS.	[47]
	rs5743818	FOLFIRI+BV (228 discovery, 297 validation)	525	Caucasian	Efficacy	Moderate	rs5743818-AA genotype: lower RR (not validated), lower PFS in discovery and validation cohorts. No significant association with PFS, OS.	[47]
*TLR7*	rs3853839	FOLFIRI+CTX (244 discovery), FOLFIRI+BV (246 control), FOLFOX/SOX+CTX (76 validation)	566	Caucasian	Efficacy	Moderate	rs3853839-GG genotype: longer PFS (validated for CTX-based) and OS (not validated). This preliminary association with PFS was not observed in BV-based cohort. No significant association with RR.	[50]
	rs187084	FOLFIRI+CTX (244 discovery), FOLFIRI+BV (246 control), FOLFOX/SOX+CTX (76 validation)	566	Caucasian	Efficacy	Moderate	rs187084-C allele: better PFS (not validated). No significant association with RR, OS.	[50]
*FPR1*	rs867228	CTX+OXA-based	1948	Caucasian	Efficacy	Moderate	No significant association with DFS, OS.	[48]
*MMP2*	rs243865	FOLFIRI+BV	486	Caucasian	Efficacy	Moderate	rs243865-any T: better OS in KRAS mutant patients.	[37]
*MAP3K7* (TAK1)	rs1145727	FOLFIRI + BV (228 discovery, 297 validation)	525	Caucasian	Efficacy	Moderate	rs1145727-AA: shorter OS (not validated). No significant association with RR, PFS.	[47]
	rs157688	FOLFIRI+BV (228 discovery, 297 validation)	525	Caucasian	Efficacy	Moderate	rs157688-CC genotype: longer PFS and OS only in univariate analyses (not validated). No significant association with RR.	[47]
	rs157432	FOLFIRI+BV (228 discovery, 297 validation)	525	Caucasian	Efficacy	Moderate	No significant association with RR, PFS, OS.	[47]
**Cytokines and chemokines in the early phase of immune response**								
*CXCL8*	rs4073	BV+FOLFOX6 (120 discovery Caucasian), FOLFOX6 (112 control Caucasian); BV-based (125 Asian); BV+FOLFOXIRI (180 Caucasian)	125–232	Mainly Caucasian	Efficacy/ Toxicity	Moderate/ Low	rs4073-A variant: shorter PFS, OS, ORR, and higher IL-8 levels only in BV-based group.	[40,51,52]
		CTX (130); 5-FU/OXA (105); Regorafenib (47); FOLFIRI+CTX (30)	30–130	Mainly Caucasian	Efficacy/ Toxicity	Low/Very low	No significant association with PFS, OS, ORR, skin rash toxicity.	[31,53,54,55]
*CXCR1/ IL8RA*	rs2234671	BV+FOLFOX/XELOX	132	Caucasian	Efficacy	Low	rs2234671-GG genotype: higher tumor RR. No significant association with PFS, OS. The combinations of CXCR1 variants may improve the prediction success for PFS and OS, and rs2234671 was the main split criteria in the decision tree for RR.	[28]
		5-FU/OXA	105	Mainly Caucasian	Efficacy	Low	rs2234671-GC genotype (vs GG): worse TTP.	[53]
		CTX ± mFOLFOX/XELOX	815	Caucasian	Efficacy/ Toxicity	Moderate	No significant association with RR, skin rash toxicity.	[33]
*CXCR2/ IL8RB*	rs2230054	BV+FOLFOX/XELOX	132	Caucasian	Efficacy	Low	rs2230054-TT: lower tumor RR. An ethnicity effect was also reported: lower RR in Caucasians but not in Asians and Hispanics. No significant association with PFS, OS.	[28]
		BV-based	125	Asian	Efficacy	Low	No significant association with ORR, PFS, OS.	[51]
		Regorafenib	47	Caucasian	Efficacy/ Toxicity	Very low	No significant association with RR, PFS, toxicity.	[54]
		5-FU/OXA	105	Mainly Caucasian	Efficacy	Low	No significant association with OS, RR, TTP.	[53]
*IL10*	rs1800896	BV-based	125	Asian	Efficacy	Low	No significant association with ORR, PFS, OS.	[51]
*IL17A*	rs2275913	BV-based	122	Caucasian	Efficacy	Low	No significant association with OS, PFS, serum cytokine levels.	[56]
*IL17F*	rs763780	BV-based	122	Caucasian	Efficacy	Low	No significant association with OS, PFS, serum cytokine levels.	[56]
**Tumor immune escape factors**								
*CD24*	rs52812045	CTX ± IRI (105 training), CTX+FOLFIRI (225)/OXA (74) (validation cohorts), BV+FOLFIRI (520 control)	924	Caucasian	Efficacy	Moderate	rs52812045-AA genotype or A allele: shorter mPFS and OS in CTX-based groups and no relation in BV-based groups.	[57]
*CTLA4*	rs231777	CTX ± IRI (105 training), CTX+FOLFIRI (225)/OXA (74) (validation cohorts), BV+FOLFIRI (520 control)	924	Caucasian	Efficacy	Moderate	rs231777-T allele and CT: higher risk of progression, and worse PFS in CTX-based group, respectively.	[57]
	rs231775	CTX ± IRI (105 training), CTX+FOLFIRI (225)/OXA (74) (validation cohorts), BV+FOLFIRI (520 control)	924	Caucasian	Efficacy	Moderate	No significant association with RR, PFS, OS.	[57]
*PDCD1* (PD1)	rs2227981; rs7421861	CTX ± IRI (105 training), CTX+FOLFIRI (225)/OXA (74) (validation cohorts), BV+FOLFIRI (520 control)	924	Caucasian	Efficacy	Moderate	No significant association with RR, PFS, OS.	[57]
*CD274* (PDL1)	rs2297137	CTX ± IRI (105 training), CTX+FOLFIRI (225)/OXA (74) (validation cohorts), BV+FOLFIRI (520 control)	924	Caucasian	Efficacy	Moderate	rs2297137-G allele: worse tumor response in CTX-based group. rs2297137-A allele had a prolonged OS in BV-based group.	[57]
	rs2297136	CTX ± IRI (105 training), CTX+FOLFIRI (225)/OXA (74) (validation cohorts), BV+FOLFIRI (520 control); OXA-based+BV (76), IRI-based+BV (65); OXA- or IRI-based + BV (152)	924; 141–152	Caucasian; Chinese	Efficacy	Low	No significant association with RR, PFS, OS in Caucasian. CD274-rs2297136-AA genotype: better PFS and OS in Chinese. In KRAS mutant Chinese subgroup, CD274-rs2297136-AA: longer PFS, trend longer OS.	[57,58,59]
	rs10122089	CTX ± IRI (105 training), CTX+FOLFIRI (225)/OXA (74) (validation cohorts), BV+FOLFIRI (520 control)	924	Caucasian	Efficacy	Moderate	No significant association with RR, PFS, OS.	[57]
KIRs/HLAs	KIR2DS4d/f	CTX-based	70	Caucasian	Efficacy	Very low	KIR2DS4 non-functional receptor homozygotes: longer OS.	[60]
	KIR haplotype combination	FOLFIRI	224	Caucasian	Efficacy	Low	Presence of haplotype combination of KIR2DL5A, 2DS5, 2DS1, 3DS1, and KIR3DS1/HLA-Bw4-I80 and absence of KIR2DS4 and 3DL1: increased CR rates.Absence of KIR2DS5 and presence of KIR3DL1/HLA-Bw4-I80: better OS.	[58]
	16 KIRs	CTX-based	70	Caucasian	Efficacy	Very low	No significant association with OS, PFS.	[60]
*ADORA2A* (A2AR)	rs5751876	FOLFIRI+BV (107 discovery), FOLFIRI+BV (215 validation), FOLFIRI+CTX (129 control)	451	Caucasian	Efficacy	Moderate	No significant association with OS, PFS, RR.	[61]
*ADORA2B* (A2BR)	rs2015353	FOLFIRI+BV (107 discovery), FOLFIRI+BV (215 validation), FOLFIRI+CTX (129 control)	451	Caucasian	Efficacy	Moderate	rs2015353-TT: longer OS in FOLFIRI+BV group.	[61]
*CD39/ ENTPD1*	rs11188513	FOLFIRI+BV (107 discovery), FOLFIRI+BV (215 validation), FOLFIRI+CTX (129 control)	451	Caucasian	Efficacy	Moderate	rs11188513-C allele: shorter PFS (not validated), OS in FOLFIRI-BV group (validated).	[61]
	rs2226163	FOLFIRI+BV (107 discovery), FOLFIRI+BV (215 validation), FOLFIRI+CTX (129 control)	451	Caucasian	Efficacy	Moderate	rs2226163-GG: longer OS only in FOLFIRI+BV group.	[61]
*CD73/ NT5E*	rs2229523	FOLFIRI+BV (107 discovery), FOLFIRI+BV (215 validation), FOLFIRI+CTX (129 control)	451	Caucasian	Efficacy	Moderate	rs2229523-A allele: longer OS in FOLFIRI+BV group.	[61]
*IDO1*	rs9657182	CTX ± IRI (105 training), CTX+FOLFIRI/OXA (225 Caucasian/ 74 Japanese validation), BV+FOLFIRI (520 control)	924	Mainly Caucasian	Efficacy	Moderate	rs9657182-CT: longer OS in CTX-based group and shortest mOS in the Japanese validation cohort. Ethnicity effect for OS. rs9657182-T allele: shorter PFS in FOLFIRI+BV group.	[57]
	rs3739319	CTX ± IRI (105 training), CTX+FOLFIRI/OXA (225 Caucasian/ 74 Japanese validation), BV+FOLFIRI (520 control)	924	Mainly Caucasian	Efficacy	Moderate	rs3739319-GG: longer mOS in CTX-based group. rs3739319-A allele: longer PFS in CTX-based group.	[57]
	rs10108662	CTX ± IRI (105 training), CTX+FOLFIRI/OXA (225 Caucasian/ 74 Japanese validation), BV+FOLFIRI (520 control)	924	Mainly Caucasian	Efficacy	Moderate	No significant association with RR, PFS, OS.	[57]
*HIF1A*	rs2057482	FOLFIRI+BV (107 discovery), FOLFIRI+BV (215 validation), FOLFIRI+CTX (129 control)	451	Caucasian	Efficacy	Moderate	No significant association with OS, PFS, RR.	[61]
	rs11549465	FOLFIRI+BV (107 discovery), FOLFIRI+BV (215 validation), FOLFIRI+CTX (129 control)	451	Caucasian	Efficacy	Moderate	No significant association with OS, PFS, RR.	[61]
*HNF1A*	rs2244608	FOLFIRI ± BV/other (167 Canadian study), FOLFIRI (250 Italian validation)	417	Caucasian	Efficacy/ Toxicity	Low	rs2244608-G: improved PFS, enhanced blood exposure to SN-38, 41% increased biliary index, 24% decreased glucuronidation ratio.	[62]
**Cytokines in the late phase of immune response**								
*IFNG*	rs2069718; rs1861493	OXA-based+BV (76), IRI-based+BV (65); OXA-based or IRI-based+BV (152)	141–152	Chinese	Efficacy	Low	No significant association with OS, PFS.	[58,59]
*IFNGR1*	rs2234711	OXA-based+BV (76), IRI-based+BV (65); OXA-based or IRI-based+BV (152)	141–152	Chinese	Efficacy	Low	rs2234711-G allele: longer OS, only in univariate analysis.	[58,59]
	rs9376267	OXA-based+BV (76), IRI-based+BV (65); OXA-based or IRI-based+BV (152)	141–152	Chinese	Efficacy	Low	rs9376267-T allele: longer OS, only in univariate analysis.	[58,59]
*IFNGR2*	rs9608753; rs1059293	OXA-based+BV (76), IRI-based+BV (65); OXA-based or IRI-based+BV (152)	141–152	Chinese	Efficacy	Low	No significant association with OS, PFS.	[58,59]
*JAK1*	rs112395617	OXA-based+BV (76), IRI-based+BV (65); OXA-based or IRI-based+BV (152)	141–152	Chinese	Efficacy	Low	No significant association with OS, PFS.	[58,59]
*JAK2*	rs1887429; rs1887428	OXA-based+BV (76), IRI-based+BV (65); OXA-based or IRI-based+BV (152)	141–152	Chinese	Efficacy	Low	No significant association with OS, PFS.	[58,59]
*STAT1*	rs3088307; rs41430444; rs6745710	OXA-based+BV (76), IRI-based+BV (65); OXA-based or IRI-based+BV (152)	141–152	Chinese	Efficacy	Low	No significant association with OS, PFS.	[58,59]
*STAT2*	rs2020854	OXA-based+BV (76), IRI-based+BV (65); OXA-based or IRI-based+BV (152)	141–152	Chinese	Efficacy	Low	No significant association with OS, PFS.	[58,59]
*IL15RA*	rs7910212	FOLFIRI (250 Italian discovery and 92 Canadian validation)	335	Caucasian	Efficacy	Moderate	rs7910212-C allele: worse OS. Highly predictive genetic score when combined with IL15RA-rs7910212-TC/CC, SMAD3-rs7179840-TT, NR1I2-rs1054190-TT, VDR-rs7299460-CC.	[46]
*SMAD3*	rs7179840	FOLFIRI (250 Italian discovery and 92 Canadian validation)	335	Caucasian	Efficacy	Moderate	rs7179840-C allele: better OS. Highly predictive genetic score when combined with IL15RA-rs7910212-TC/CC, SMAD3-rs7179840-TT, NR1I2-rs1054190-TT, VDR-rs7299460-CC.	[46]
*CCL3*	rs1130371	Regorafenib (79 Japan discovery, 150 Italian validation)	229	Japan and Italian	Efficacy/ Toxicity	Low	rs1130371-A allele: shorter PFS (in Japan and Italian cohort) and OS (only in validation Italian cohort). Ethnicity effect for OS. rs1130371-GG variant: higher CCL5 serum level changes between baseline and day 21 but did not correlate with CCL3 levels.	[63]
		FOLFOX+BV (61 evaluation), FOLFOX/XELOX+BV (71 validation), FOLFOX (84 control)	216	Asian	Efficacy	Low	No significant association with PFS, OS, RR.	[64]
		Regorafenib (79 Japan discovery, 150 Italian validation)	229	Japan and Italian	Efficacy (serum level)	Low	rs1130371-GG genotype: increased CCL3 levels at day 21.	[65]
*CCL4*	rs1634517	Regorafenib (79 Japan discovery, 150 Italian validation)	229	Japan and Italian	Efficacy/ Toxicity	Low	rs1634517-A allele: shorter PFS (in Japan and Italian cohort), OS (only in Italian validation cohort). rs1634517-CC variant: higher CCL5 serum level changes at baseline and day 21 but did not correlate with CCL4 levels.	[63]
		FOLFOX+BV (61 evaluation), FOLFOX/XELOX+BV (71 validation), FOLFOX (84 control)	216	Asian	Efficacy	Low	rs1634517-A allele: shorter OS (only in control group treated without BV).	[64]
		Regorafenib (79 Japan discovery, 150 Italian validation)	229	Japan and Italian	Efficacy (serum level)	Low	rs1634517-CC variant: higher CCR5 changes between baseline and day 21. Pattern of decreased CCL4 levels at day 21 had a trend toward longer PFS.	[65]
*CCL5*	rs2280789	Regorafenib (79 Japan discovery, 150 Italian validation)	229	Japan and Italian	Efficacy/ Toxicity	Low	rs2280789-GG: longer OS (only in Japan discovery cohort), higher incidence of grade>3 hand–foot skin reaction, lower CCL5 level at baseline and day 21, lower VEGF-A level at day 21. rs2280789 and rs3817655 showed high LD.	[63]
		FOLFOX+BV (61 evaluation), FOLFOX/XELOX+BV (71 validation), FOLFOX (84 control)	216	Asian	Efficacy	Low	rs2280789-GG: shorter OS (in control group treated without BV). rs2280789-G allele: longer PFS, OS, RR in FOLFOX+BV compared FOLFOX. rs2280789-G allele: higher VEGF-A level at baseline, greater decrease of VEGF-A levels at day 14 and 56.	[64]
		FOLFIRI+CTX (244 evaluation), FOLFIRI+BV (247 control)	491 *KRAS* wt	Caucasian	Efficacy	Moderate	rs2280789-G allele: shorter OS. Combined with tumor location (left/right) a better stratification emerged: left- rs2280789-AA better mOS, right-rs2280789-G allele worse mOS.	[66]
		Regorafenib (79 Japan discovery, 150 Italian validation)	229	Japan and Italian	Efficacy (serum level)	Low	rs2280789-G allele: higher CCL3 level between baseline and day 21, but CCL4 decreased.	[65]
	rs3817655	Regorafenib (79 Japan discovery, 150 Italian validation)	229	Japan and Italian	Efficacy/ Toxicity	Low	rs3817655-TT: longer OS (only in Japan discovery cohort), higher incidence of grade>3 hand–foot skin reaction, lower CCL5 level at baseline and day 21, lower VEGF-A level at day 21. rs2280789 and rs3817655 showed high LD.	[63]
		Regorafenib (79 Japan discovery, 150 Italian validation)	229	Japan and Italian	Efficacy (serum level)	Low	rs3817655-TT: higher CCL3 level between baseline and day 21, but CCL4 decreased. Increased CCL3 level at PD was associated with longer OS.	[65]
*CCR5*	rs1799988	Regorafenib (79 Japan discovery, 150 Italian validation)	229	Japan and Italian	Efficacy/ Toxicity	Low	rs1799988-TT variant: higher risk of no DC (PD), higher CCL5 serum level changes at baseline and day 21. No significant association with OS, PFS.	[63]
		FOLFOX+BV (61 evaluation), FOLFOX/XELOX+BV (71 validation), FOLFOX (84 control)	216	Asian	Efficacy	Low	rs1799988-T allele: shorter OS (only in control group without BV). CCR5 rs1799988-T allele: shorter OS.	[64]
		FOLFIRI+CTX (244 evaluation), FOLFIRI+BV (247 control)	491 *KRAS* wt	Caucasian	Efficacy	Moderate	rs1799988-T allele: lower RR (trend), shorter PFS (only in evaluation). Opposite effects between right- and left-sided tumors: TT variant: favorable in right-sided tumors, while T allele was unfavorable in left-sided tumors for tumor response, PFS, and OS.	[66]
		Regorafenib (79 Japan discovery, 150 Italian validation)	229	Japan and Italian	Efficacy (serum level)	Low	Patients with TS had lower mean changes in serum CCR5 levels between baseline and day 21. No significant differences in DC and TS at baseline, higher in TS at day 21.	[65]
*CDX2*	rs3812863	FOLFOX ± BV (146 Japan discovery), FOLFOXIRI+BV (230 Caucasian validation), FOLFIRI+BV (228 Caucasian control)	604	Japan and Caucasian	Efficacy	Moderate	rs3812863-GG genotype: higher ORR, trend of better OS, PFS.	[67]
*MS4A12*	rs4939378	FOLFOX ± BV (146 Japan discovery), FOLFOXIRI+BV (230 Caucasian validation), FOLFIRI+BV (228 Caucasian control)	604	Japan and Caucasian	Efficacy	Moderate	rs4939378-GG genotype: longer OS, PFS.	[67]
*HIF1A*	rs12434438	Regorafenib (79 Japan discovery, 150 Italian validation)	229	Japan and Italian	Efficacy/ Toxicity	Low	No significant association with PFS, OS, toxicity.	[63]
**Regulated cell death factors**								
*FCGR2A*	rs1801274	CTX-based	39–1123	Mainly Caucasian	Efficacy	High	Meta-analysis: rs1801274-His (A allele): generally, no benefit for mOS, mORR, mDCR in CTX-based regimen.	[31,60,68,69,70,71,72,73,74,75,76,77,78,79,80,81,82,83,84,85,86]
*FCGR3A*	rs396991	CTX-based	39–2831	Mainly Caucasian	Efficacy/ Toxicity	High	Meta-analysis: rs396991-Phe/Phe (AA genotype): longer OS, PFS, and PFS in *KRAS* wt.	[31,60,68,69,70,71,72,73,74,75,76,77,78,79,80,81,82,83,84,85,86]
*ANXA1*	rs1050305	BV+FOLFOX (161 discovery), BV+FOLFOXIRI (109 validation), BV+FOLFIRI (378 control)	648	Caucasian	Efficacy	Moderate	rs1050305-G allele: worse OS (validated), PFS, RR only in BV+FOLFOXIRI group. No significant association with PFS, OS, ORR in BV+FOLFIRI group.	[87]
*CALR*	rs1010222	BV+FOLFOX (161 discovery), BV+FOLFOXIRI (109 validation), BV+FOLFIRI (378 control)	648	Caucasian	Efficacy	Moderate	rs1010222-A allele: better PFS (not validated). No significant association with PFS, OS, ORR in BV+FOLFIRI group.	[87]
	rs1049481	BV+FOLFOX (161 discovery), BV+FOLFOXIRI (109 validation), BV+FOLFIRI (378 control)	648	Caucasian	Efficacy	Moderate	No significant association with PFS, OS, ORR.	[87]
*ATG4B*	rs35271226; rs1130910; rs7421; rs34691302	OXA-based (188), IRI-based (137)	325	Chinese	Efficacy	Low	No significant association with OS, PFS, DCR.	[88]
*ATG16L1*	rs6758317; rs2241878; rs7595748	OXA-based (188), IRI-based (137)	325	Chinese	Efficacy	Low	No significant association with OS, PFS, DCR.	[88]
*ATG2B*	rs17094017	OXA-based (188), IRI-based (137)	325	Chinese	Efficacy	Low	rs17094017-T allele (additive model): increased OS, PFS, DCR in overall population. rs17094017-T allele (additive model): increased OS, PFS, DCR in OXA-based not in IRI-based group (stratifying for chemotherapy).	[88]
	rs8019013	OXA-based (188), IRI-based (137)	325	Chinese	Efficacy	Low	rs8019013-T allele (additive model): shorter PFS, prolonged DCR in univariate, not confirmed after FDR correction.	[88]
	rs12432561; rs10134160	OXA-based (188), IRI-based (137)	325	Chinese	Efficacy	Low	No significant association with OS, PFS, DCR.	[88]
*ATG13*	rs13448	FOLFIRI+BV or FOLFIRI+CTX	657	Chinese	Toxicity	Moderate	rs13448-any C: lower rate of grade 2–3 hypertension	[89]
*FIP200*	rs1129660	FOLFIRI+BV or FOLFIRI+CTX	657	Chinese	Toxicity	Moderate	rs1129660-any G: lower rate of grade 2 or 3 hypertension	[89]
*ULK1*	rs9481	FOLFIRI+BV or FOLFIRI+CTX	657	Chinese	Toxicity	Moderate	rs9481-A allele: lower rate of grade 2 or 3 hypertension	[89]
*GABARAPL2*	rs11149841; rs6564267	OXA-based (188), IRI-based (137)	325	Chinese	Efficacy	Low	No significant association with OS, PFS, DCR.	[88]
*WIPI1*	rs11658979; rs11077558; rs2011143; rs2909207; rs883622; rs883620; rs35271226	OXA-based (188), IRI-based (137)	325	Chinese	Efficacy	Low	No significant association with OS, PFS, DCR.	[88]

**Abbreviations**: CT: chemotherapy, BV: bevacizumab, CTX: cetuximab, PANI: panitumumab, FL: fluoropyrimidines, OXA: oxaliplatin, Cape: capecitabine; 5-FU: 5-fluoropyrimidine, IRI: irinotecan, SOX: oxaliplatin + S-1 regimen, (m)OS: (median) overall survival; (m)PFS: (median) progression-free survival; (O)RR: (overall) response rate; ADCC: antibody-dependent cellular cytotoxicity; DCR: disease control rate; PD: progression; TS: tumor shrinkage; CR: complete response; LD: linkage disequilibrium; N: number of patients; Ref.: references. 3. Acute-phase cytokines and enzymes.

**Table 2:** Final summary with high-level evidence for efficacy and toxicity focusing on therapy.

Therapy	Genes	rs Code/Alias	Allele/Genotype	Clinical Outcome
**CETUXIMAB**	*STING1*	rs7380824	T allele	Worse ORR
		rs1131769	T allele	Worse OS
	*IFNB1*	rs1051922	GA, AA genotypes	Worse PFS
	*TLR7*	rs3853839	GG genotype	Better PFS
	*CD24*	rs52812045	A allele	Worse PFS, OS
	*CTLA-4*	rs231777	T allele	Worse PFS
	*CD274*	rs2297137	G allele	Worse ORR
	*IDO1*	rs9657182	CT genotype	Better OS in Caucasian Worse OS in Japanese
		rs3739319	GG genotype	Better OS
	*CCL5*	rs2280789	G allele	Worse OS in *KRAS* wild-type
	*CCR5*	rs1799988	T allele	Worse PFS, RR
	*FCGR3A*	rs396991	C allele	Better OS, PFS, and PFS in *KRAS* wild-type
**BEVACIZUMAB**	*IL6*	rs2069837	G allele	Worse PFS, ORR
	*CXCR4*	rs2228014	T allele	Worse PFS, OS
	*CCL2*	rs4586	C allele	Better PFS in *KRAS* mutant
	*CXCL8*	rs4073	A allele	Worse PFS, OS, ORR
	*TBK1*	rs7486100	T allele	Worse PFS in *KRAS* wild-type
	*IRF3*	rs2304205	C allele	Better PFS in *KRAS* mutant
	*CD274*	rs2297137	G allele	Better OS
	*ADORA2B*	rs2015353	TT genotype	Better PFS
	*CD39/ENTPD1*	rs11188513	C allele	Worse OS
		rs2226163	GG genotype	Better OS
	*CD73/NT5S*	rs2229523	A allele	Better OS
**FOLFIRI + BEVACIZUMAB**	*TLR1*	rs5743618	TT genotype	Worse RR
	*TLR6*	rs5743818	AA genotype	Worse PFS
	*IDO1*	rs9657182	T allele	Worse PFS
	*NOS2*	CCTTT repeat	>13 repeats	Better PFS
**FOLFIRI**	*STAT-3*	rs1053004	C allele	Lower toxicity
	*IGF1*	rs2946834	A allele	Better PFS; PFS, OS in RAS wild-type
	*IRS1*	rs1801123	C allele	Worse OS; OS in RAS wild-type
	*NR1L2/PXR*	rs1054190	TT genotype	Worse OS
	*NR1L1/VDR*	rs7299460	T allele	Better OS
		rs11574077	G allele	Higher toxicity
	*IL15RA*	rs7910212	C allele	Worse OS
	*SMAD3*	rs7179840	C allele	Better OS
**OXALIPLATIN**	*CDX2*	rs3812863	GG genotype	Better ORR, OS, PFS
	*MS4A12*	rs4939378	GG genotype	Better OS, PFS
	*ANXA1*	rs1050305	G allele	Worse OS
